# Tailoring Structure–Property Relationships in π‐Conjugated Heterocyclic Poly(arylene alkylene) Anion‐Exchange Membranes for High‐Performance Water Electrolysis

**DOI:** 10.1002/advs.77491

**Published:** 2026-08-31

**Authors:** Qian Wang, Riyang Huang, Wenzhe Zhao, Si Chen, Yunpeng Guo, Yang Wu, Yun Zhao, Patric Jannasch, Jingshuai Yang

**Affiliations:** ^1^ Department of Chemistry College of Sciences Northeastern University Shenyang China; ^2^ Fuel Cell System and Engineering Laboratory Key Laboratory of Fuel Cell & Hybrid Power Sources Dalian Institute of Chemical Physics Chinese Academy of Sciences Dalian China; ^3^ Polymer & Materials Chemistry Department of Chemistry Lund University Lund Sweden; ^4^ College of Chemistry Liaoning University Shenyang Liaoning China

**Keywords:** anion exchange membrane, copolymer, ion transport mechanism, water electrolysis

## Abstract

Anion‐exchange membrane water electrolysis (AEMWE) is a promising technology for sustainable hydrogen production, but practical applications are limited by the trade‐off between hydroxide conductivity and alkaline stability of anion‐exchange membranes (AEMs). Here, we report a molecular design strategy that regulates ion transport pathways and membrane stability by tethering quaternary ammonium cations via flexible side chains to π‐conjugated heterocyclic backbone polymers. Durable cationic copolymers containing *p*‐terphenyl with dibenzofuran (DBF) or dibenzothiophene (DBT) units are synthesized. Combined experimental and theoretical studies establish structure‐property relationships linking heteroatom chemistry to the hydration, microphase morphology, and ion transport. DBF‐units promote dense hydrogen bonding networks, whereas DBT‐units strengthen ion‐dipole interactions and induce more pronounced microphase separation. Consequently, optimized DBF‐ and DBT‐based membranes exhibit hydroxide conductivities exceeding 180 and 200 mS cm^−1^, respectively, at 80°C. In AEMWEs using non‐precious‐metal catalysts, these membranes deliver current densities above 4.1 and 5.1 A cm^−2^ at 2 V, respectively. The DBT‐based membrane also maintains stable operation for over 1600 h at 1 A cm^−2^ and 60°C. This work establishes structure‐performance relationships and provides a practical molecular design strategy for highly conductive, durable AEMs based on π‐conjugated heterocyclic backbone units.

## Graphical abstract

From passive to active ion transport: π‐conjugated heterocycles reconfigure the hydration environment and microphase morphology of *p*‐terphenyl‐based anion‐exchange membranes, enabling fast hydroxide transport and high‐performance water electrolysis.

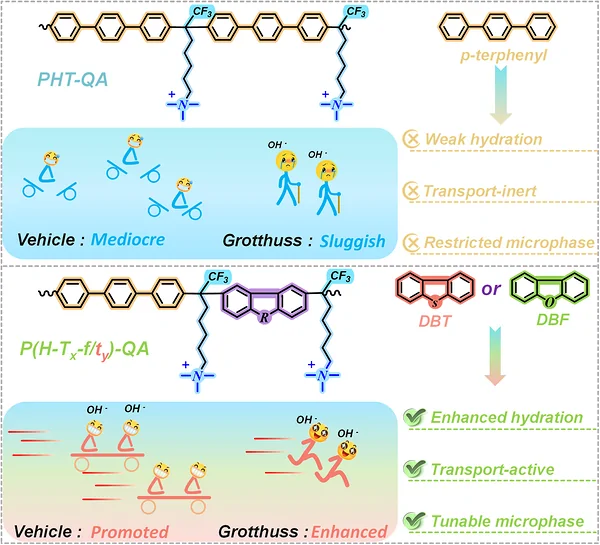

## 1 Introduction

The intensifying climate crisis and the dependence on fossil fuel resources are accelerating the transition toward renewable energy systems based on hydrogen, wind, and solar power. Water electrolysis provides an effective route to convert intermittent renewable electricity into storable chemical energy in the form of hydrogen [1, 2]. Among the available technologies, anion exchange membrane water electrolysis (AEMWE) has attracted increasing attention owing to its unique advantages [3, 4, 5]. Operating under alkaline conditions, AEMWE enables the use of non‐precious metal catalysts and does not require expensive titanium bipolar plates, as the environment is less corrosive than that in acidic systems [3, 6]. Compared with proton exchange membrane water electrolysis (PEMWE), AEMWE offers a more cost‐effective solution [5, 7], while also providing a simpler system configuration and improved operational safety relative to conventional alkaline water electrolysis (ALK), together with higher gas purity [8, 9]. These features position AEMWE as a key technology for the development of a sustainable hydrogen economy.

The anion exchange membrane (AEM) plays a central role in AEMWE and must simultaneously address two fundamental challenges: the intrinsically lower mobility of hydroxide ions compared to protons [10], and the limited chemical stability of cationic groups under strongly alkaline conditions [8, 11, 12]. Achieving a balance between high hydroxide conductivity and long term alkaline durability therefore imposes stringent requirements on polymer design. Early AEMs commonly employed benzylic quaternary ammonium (QA) groups, which provided high ionic conductivity but suffered from rapid degradation in strongly alkaline conditions [12, 13, 14]. Structural modifications that eliminate aryl ether linkages and benzyl groups have been shown to significantly improve alkaline stability. For instance, Jannasch and co‐workers developed ether‐free poly(arylene piperidine)‐based AEMs (PAPipQs) incorporating piperidinium cations into the main chain, where the conformational restriction from the cyclic structure enhances resistance to alkaline degradation [15]. However, their hydroxide conductivity remains lower than that of AEMs functionalized with trimethyl alkyl QA groups [16, 17, 18]. For example, a poly(isatin biphenylene)‐based AEM bearing QA groups (QAPIB) achieved a hydroxide conductivity of 94 mS cm^−1^ at 80°C [17], significantly higher than those of analogous membranes functionalized with pyrrole (49 mS cm^−1^) and piperidine (43 mS cm^−1^) under identical conditions.

Decoupling the cationic groups from the polymer backbone through the introduction of flexible alkyl spacers represents an effective strategy to enhance membrane performance [19]. Long alkyl chains reduce the electron‐withdrawing effect of cationic groups on the backbone, thereby improving chemical stability. At the same time, their flexibility allows the cationic moieties to reorient and form dynamic hydrogen bonding networks that facilitate Grotthuss type ion transport [20, 21, 22]. In addition, such side chains promote nanoscale phase separation, leading to the formation of continuous ion‐conducting channels [21, 22, 23, 24]. The spacer length is a critical parameter, as an appropriate chain length can enhance ion mobility while ensuring dimensional stability [25, 26]. For example, Zhang et al. reported on a multiblock copolymer AEM (PBPA‐*b*‐BPP‐0.10) with cations tethered via heptyl spacers [27], which exhibited an OH^−^ conductivity of 162 mS cm^−1^ at 80°C and retained 86.6% of its QA groups after 3750 h in 2 m NaOH. Nevertheless, AEMs with long side chains have not yet reached optimal conductivity, possibly due to insufficient polarity within the hydrophobic domains.

Another effective approach to improving AEM performance involves tailoring the polymer backbone through copolymerization and rational monomer selection [11, 23, 28]. Terphenyl units are widely used in superacid catalyzed polymerizations, producing polymers with excellent mechanical strength and chemical stability [16, 29, 30]. However, their non‐polar nature limits their contribution to ion transport. To address this limitation, we have previously incorporated dibenzo‐18‐crown‐6 into the polymer backbone, where its ability to complex with K^+^ ions and promote phase separation resulted in enhanced conductivity and alkaline stability [31]. More recently, dibenzocyclic heteroaromatic units, such as dibenzofuran [32, 33, 34], have been introduced as main‐chain building blocks. The lone pair of electrons of the heteroatoms and the delocalized π‐electron systems facilitate hydrogen‐bond formation and promote microphase separation, while the rigid planar structure enhances chemical robustness. A similar strategy has been applied using dibenzothiophene [35, 36]. For example, Sun et al. and our group separately incorporated dibenzothiophene into a poly(terphenyl piperidine) backbone to construct high‐performance copolymer AEMs [36, 37] where the sulfur atom contributes to a well‐distributed hydrogen bonding network. The resulting membrane exhibited a hydroxide conductivity of 182 mS cm^−1^, significantly exceeding that of the terphenyl‐based PAP‐TP‐100 (108 mS cm^−1^), and enabled stable operation at 2 A cm^−2^ for 650 h at 40°C [36]. These results highlight the considerable potential of dibenzocyclic aromatic units as functional building blocks for advanced AEM design.

**SCHEME 1:**
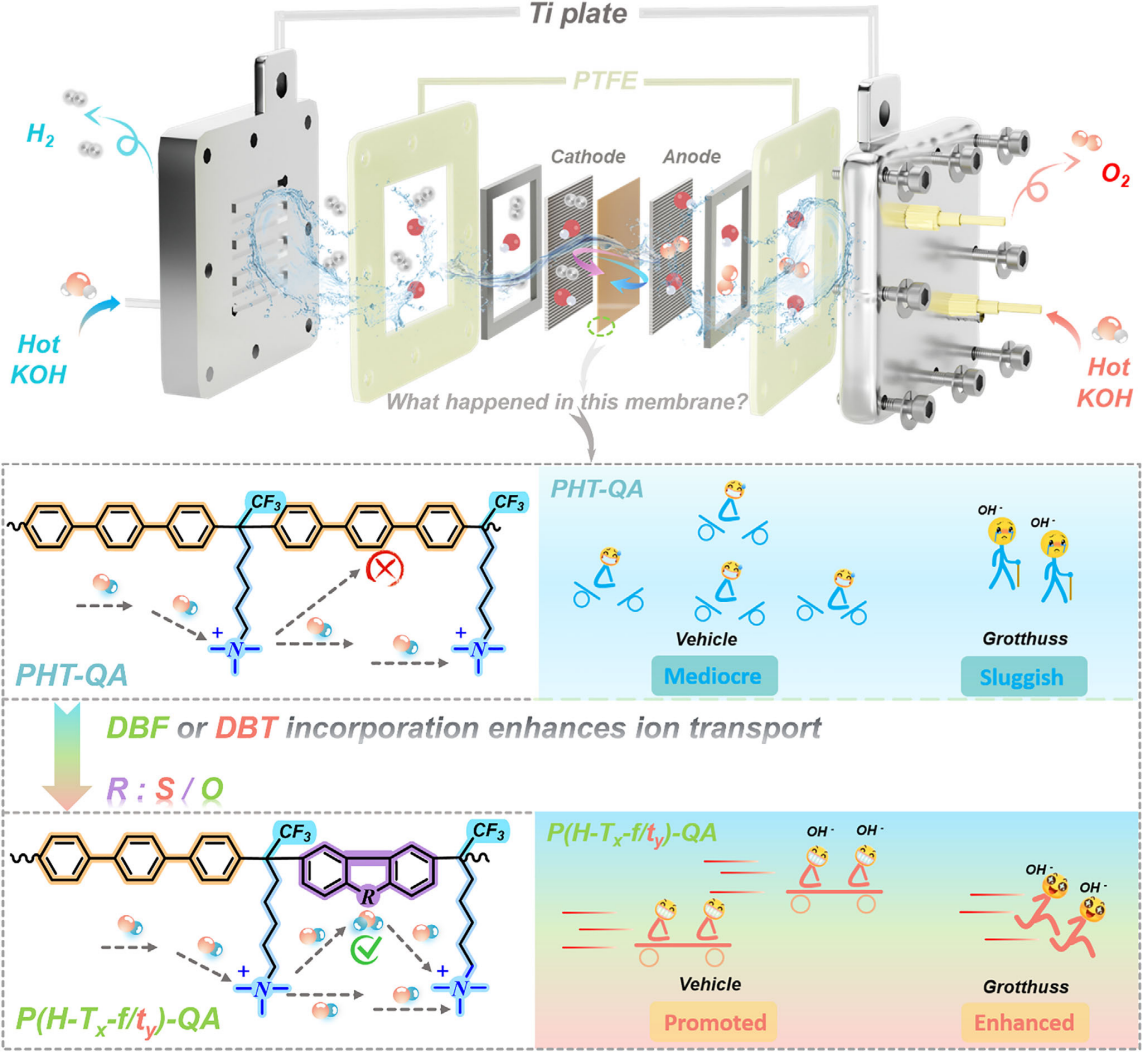
Molecular design concept and proposed ion‐transport characteristics of the present AEMs. The incorporation of π‐conjugated heteroaromatic units (DBF/DBT) and long flexible alkyl side chains optimizes the ion‐transport behavior and therefore enhances hydroxide conductivity.

In the present work, two π‐conjugated monomers, dibenzofuran (DBF) and dibenzothiophene (DBT), were introduced as main‐chain components in poly(arylene alkylene)s via Friedel‐Crafts polyhydroxyalkylations with *p*‐terphenyl and a long‐chain functional monomer, 7‐bromo‐1,1,1‐trifluoro‐2‐heptanone, as shown in Figure S1. This strategy afforded a series of AEMs, denoted as P(H‐T*_x_*‐f*_y_*) and P(H‐T*_x_*‐t*_y_*), where “H” represents the heptyl bromide functionality, and *x* and *y* correspond to the molar ratios of terphenyl and DBF or DBT units, respectively. The incorporation of heteroatoms containing lone‐pair electrons is expected to modulate the local hydration environment and hydrogen bonding network, thereby influencing the hydroxide‐ion transport within the membrane. Meanwhile, the intrinsic polarity and extended π‐conjugation of DBF and DBT units can promote microphase separation and increase free volume, both of which are beneficial for hydrated ion transport. Importantly, the differences in electronegativity and electronic structure between oxygen and sulfur impart different physicochemical characteristics to DBF‐ and DBT‐based membranes. We hypothesized that these differences would result in distinct hydration environments and ion‐transport mechanism, as illustrated in Scheme 1. By systematically varying the monomer composition, a series of copolymer membranes were prepared to elucidate structure‐property relationships through a combination of experimental characterization and theoretical calculations. This study ultimately identifies a new class of AEMs that achieves a balance between high conductivity, robust alkaline stability, and structural integrity, providing valuable insights into the molecular design of high‐performance membranes for alkaline water electrolysis.

## 2 Results and Discussion

### 2.1 Chemical Structures and Structure Simulation

**FIGURE 1:**
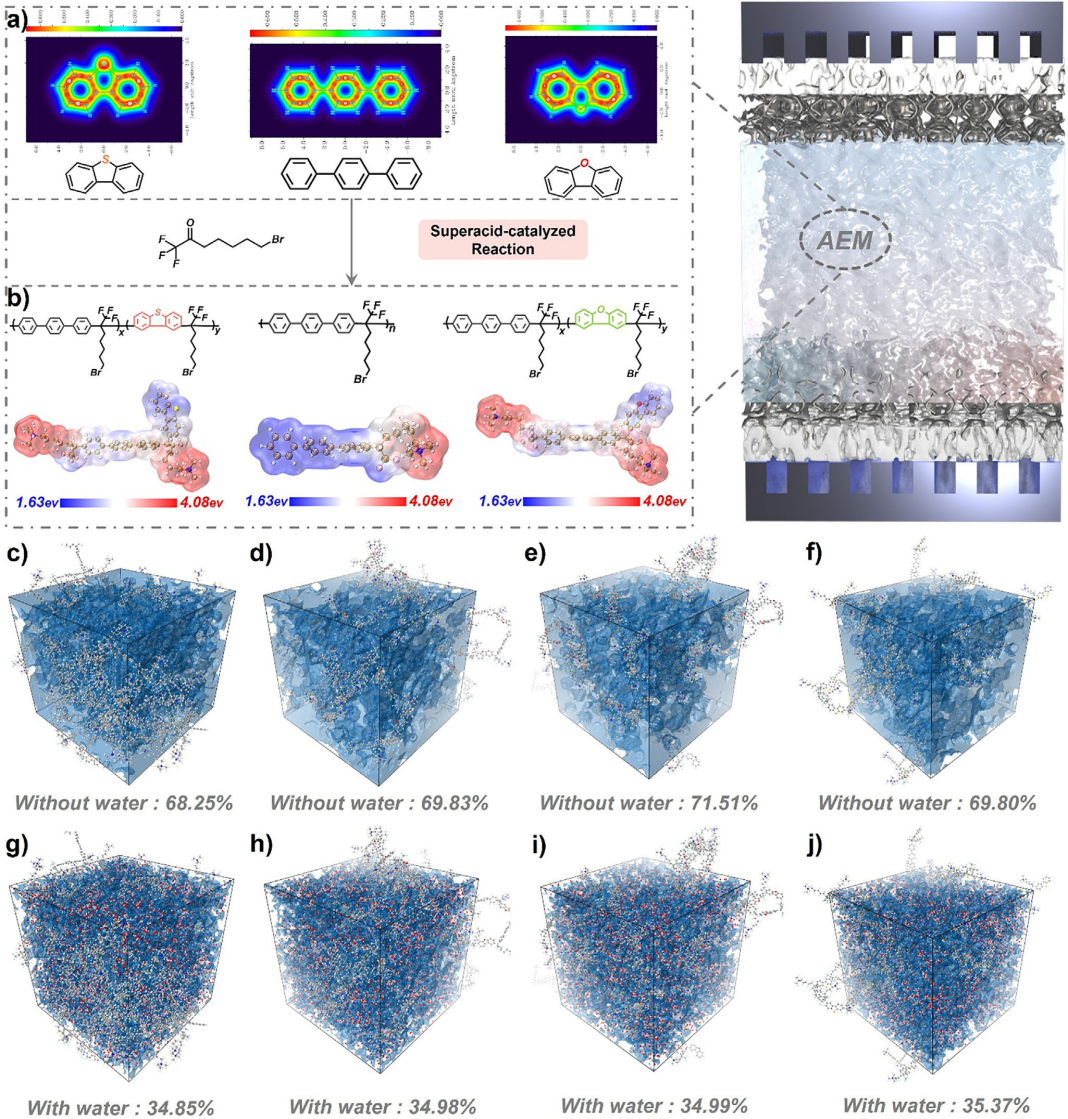
a) The conjugation characteristics of the aromatic compounds, b) ESP of the polymer repeating units; MD simulation results of PHT‐QA, P(H‐T_53%_‐f_47%_)‐QA, PHf‐QA and P(H‐T_51%_‐t_49%_)‐QA membranes in the dry c–f) and wet g–j) state.

To elucidate the influence of chemical structure on monomer reactivity in superacid catalyzed polyhydroxyalkylations, the electronic structures and orbital characteristics of the aromatic monomers were first analyzed. In DBT, the sulfur atom adopts an sp^2^ hybridization state, with its lone pair of electrons occupying p orbitals perpendicular to the molecular plane. This configuration enables effective overlap with the π system, resulting in enhanced electron delocalization. This is supported by the shortened C─S bond length (1.76 Å) compared to a typical C─S single bond (1.82 Å), indicating significant conjugation involving the sulfur lone pair. In contrast, for DBF, one lone pair of the oxygen atom resides in a p orbital, while the other occupies an sp^2^ hybrid orbital. The asymmetry in orbital distribution leads to a less effective overlap with the π system. In addition, the high electronegativity of oxygen limits the participation of its lone pair of electrons in conjugation, resulting in a predominantly inductive electron donating effect (Figure 1a). These fundamental electronic differences highlight the distinct reactivity of DBF and DBT in superelectrophilic Friedel‐Crafts polyhydroxyalkylation reactions. To quantify reactivity, a dual descriptor analysis was performed (Figure S2). Both DBT and DBF exhibit significantly lower dual descriptor values (Δ*f* = ‐0.0329 and ‐0.0115, respectively) than *p*‐terphenyl (‐0.0021), indicating higher electrophilic reactivity. In general, increased monomer reactivity will accelerate polymerization kinetics and promote chain growth, resulting in higher molecular weights under identical reaction conditions. However, for the DBT‐containing polymers, the rapid polymerization was regulated by shortening the reaction time to avoid uncontrolled chain growth and possible side reactions, thereby maintaining a molecular weight comparable to those of the other polymers. Still, the synthesis of the pure DBT‐based homopolymer (i.e., PHt) was unsuccessful because of the very rapid and uncontrollable polymerization process. These results suggest that the conjugated heteroaromatic structures facilitate electrophilic substitution and polymer growth while requiring careful regulation of reaction kinetics to achieve controllable polymer architectures. Furthermore, the rigid aromatic frameworks introduce distinct electrostatic potential (ESP) distributions within the polymer repeating units, which influence intermolecular interactions and segmental dynamics (Figure 1b). These structural features are expected to play a critical role in determining the ion transport behavior in the resulting AEMs (Scheme 1).

The chemical structures of all polymers, before and after quaternization, were confirmed by ^1^H NMR spectroscopy. As shown in Figure S3, for pristine PHT, the electron‐withdrawing trifluoromethyl group decreases the electron density on adjacent aromatic rings, resulting in downfield shifts of proton signals to 7.35–7.80 ppm [27]. In the DBF‐containing polymers (PHf), the electron‐withdrawing effect of the oxygen atoms further reduces electron density, giving rise to characteristic signals at 7.80–8.10 ppm, which are assigned to the aromatic protons of DBF [32, 37]. In the side chains, the methylene groups of the trifluorobromoheptyl substituents display distinct chemical shifts due to the combined influence of the trifluoromethyl and bromine groups. The strong electron‐withdrawing effect of CF_3_ causes pronounced downfield shifts for the nearby methylene protons, while the effect of bromine is weaker and declines more rapidly along the chain. Copolymer compositions were determined by integrating aromatic and methylene signals, confirming the successful synthesis of a series of DBF‐ and DBT‐based copolymers with controlled compositions. As shown in Figures S4 and S5, a new signal at approximately 3.00 ppm appears after quaternization, corresponding to the methyl protons of the QA groups [27, 32]. The degree of quaternization, calculated from the integration of these signals according to Equation S1, reaches 95–99% for all samples, indicating nearly complete functionalization. These results confirm the successful synthesis and modification of the target polymers.

Further structural verification was obtained by FTIR and XPS analyses. The FTIR spectra (Figures S6 and S7) show a strong absorption at 1145 cm^−1^, attributed to C─F stretching [38], confirming the presence of the trifluoromethyl groups. The DBT‐containing copolymers exhibit characteristic C─S vibrations, while DBF‐based polymers show distinct C─O stretching signals [37], enabling a clear differentiation between the two structures. After quaternization, the appearance of a new band at approximately 905 cm^−1^, assigned to C─N stretching, confirms the successful introduction of QA groups. XPS measurements provide further insight into the elemental composition and bonding environments, as shown in Figures S8–S11. The C─F bond exhibits a pronounced shift to higher binding energy (292.5 eV), reflecting the strong electron‐withdrawing effect of fluorine (Figure S9a). In contrast, the C─Br bond shows a smaller shift due to the lower electronegativity of bromine [39]. For DBT‐containing polymers, characteristic S 2p peaks corresponding to spin–orbit splitting are observed at 163.9 and 164.1 eV (S 2p_3/2_ and 2p_1/2_, Figure S11b) [40], confirming the presence of sulfur in the heterocyclic structure. Quaternization is further evidenced by the disappearance of the C─Br signal and the emergence of C─N bonds (Figure S9b), along with the detection of ionic bromide species (Figure S10b) [39]. The N 1s spectra display peaks corresponding to QA groups and residual amine species (Figure S11a) [31]. These combined results provide further evidence for the successful synthesis and functionalization of the copolymer AEMs.

**FIGURE 2:**
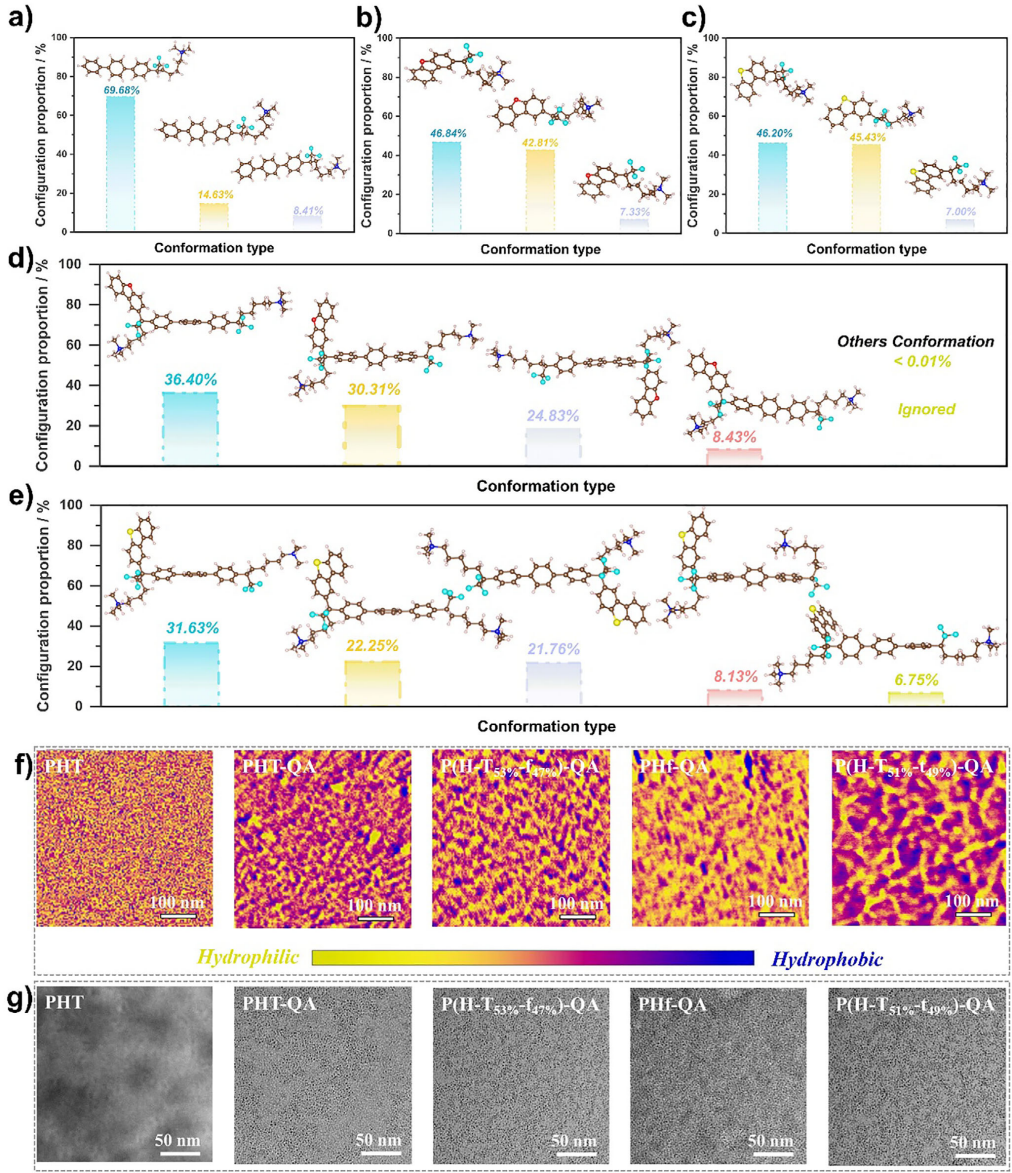
Boltzmann distribution probabilities of different conformations of the periodic units of polymers a) PHT‐QA, b) PHf‐QA, c) PHt‐QA, d) P(H‐T_x_‐f_y_)‐QA, e) P(H‐T_x_‐t_y_)‐QA; f) AFM and g) TEM phase images of selected key polymers.

The conformational behavior of the polymers was further investigated by density functional theory (DFT) calculations. Due to the presence of the flexible heptyl side chains, the polymers can adopt multiple conformations; however, only a limited number of low‐energy structures dominate under thermodynamic equilibrium [41]. The optimized structures reveal that DBF‐ and DBT‐containing polymers exhibit more twisted backbones compared to the relatively planar PHT structure (Figure 2a–e). This difference suggests a reduced chain packing density and increased free volume in the copolymers. To evaluate the alkaline stability, frontier molecular orbital energies and electrostatic potential (ESP) maps were analyzed. Degradation of AEMs in alkaline environments is typically initiated by nucleophilic attack of hydroxide ions, involving electron transfer to the lowest unoccupied molecular orbital (LUMO). Therefore, higher LUMO energy levels are generally associated with improved resistance to alkaline degradation [31, 37, 42]. As shown in Figure S12, the calculated results show that the DBF‐ and DBT‐based polymers possess LUMO energies comparable to each other, but slightly lower than that of PHT. However, LUMO energy alone does not fully determine the stability. ESP analysis reveals localized electron‐rich regions near the QA side chains in the copolymers. This electron delocalization reduces local polarity and may suppress the presence of hydroxide [31], thereby mitigating degradation. These findings suggest that the incorporation of DBF and DBT units provides a protective electronic environment for the cationic groups, contributing to enhanced alkaline stability.

X‐ray diffraction (XRD) analysis was used to evaluate the chain packing of the polymers (Figure S13). The inter‐chain spacing, calculated from the amorphous diffraction peaks, increases from 4.88 Å in PHT to 5.14 Å in DBF‐rich copolymers, reflecting the influence of steric hindrance and backbone distortion [32, 36]. After quaternization, electrostatic repulsion between cationic groups further expands the spacing, indicating increased free volume. Molecular dynamics (MD) simulations were conducted to quantify the free volume fraction (FFV) and visualize ion transport pathways. As shown in Figure 1c–f and Figure S14a, both the PHf‐QA and the PHt‐QA membranes exhibit higher FFV values (71.51% and 71.17%, respectively) than PHT (68.25%) under dry conditions, due to their twisted conformations [37]. Upon hydration, water molecules partially occupy the free volume cavities, resulting in a reduction of the detectable void fraction (Figure 1g–j, Figure S14b, and Table S1). The FFV values were 34.85%, 34.99% and 35.23% for hydrated PHT‐QA, PHf‐QA and PHt‐QA membranes, respectively. The more pronounced decrease in FFV for hydrated PHf‐QA and PHt‐QA is attributed to the stronger hydration capability of the DBF and DBT units, whose heteroatoms act as additional hydrogen‐bonding acceptors and promote higher water uptake and pore filling. Notably, the copolymer P(H‐T_50%_‐t_50%_)‐QA retains the highest effective FFV (35.37%) under hydrated conditions. This observation is attributed to a favorable balance between hydration‐induced pore filling and chain packing disruption arising from the coexistence of terphenyl and DBT units, also suggesting a favorable balance between structural rigidity and segmental mobility. These results demonstrate that the combination of π‐conjugated heteroaromatic units (especially DBT) and flexible long alkyl side chains effectively enhances free volume and facilitates the formation of ion‐conducting pathways.

### 2.2 Morphology and Physicochemical Properties

As shown in Figure S15, the appearance of the membranes changed from opaque yellow to clearly transparent after quaternization, indicating improved homogeneity and uniformity. Scanning electron microscopy shows that all membranes possess smooth, dense, and pore‐free surfaces, which are essential for suppressing gas crossover and ensuring high hydrogen purity in AEMWEs [23, 29]. The microphase morphology was further investigated by atomic force microscopy (AFM) and transmission electron microscopy (TEM) (Figure 2f,g and Figure S15). In the AFM phase images, hydrophilic domains appear bright, whereas hydrophobic regions appear dark. The PHT membrane exhibits a relatively uniform morphology without clear phase separation, which is attributed to its hydrophobic backbone structure. In contrast, the P(H‐T*_x_*‐f*_y_*) copolymers display increasingly pronounced microphase separation with increasing DBF content. This observation arises from the difference in polarity and hydrophilicity between the DBF and terphenyl units, promoting the phase segregation [3, 14]. As a result, PHf exhibits larger hydrophilic domains compared to PHT. Quaternization further amplifies this effect by introducing strongly polar and hydrophilic QA groups, leading to more distinct phase separated morphologies. Notably, DBT containing membranes such as P(H‐T_51%_‐t_49%_)‐QA exhibit extended, percolating and channel‐like phase domains, which are highly favorable for efficient ion transport. TEM observations are consistent with these findings. While PHT‐QA contains densely arranged hydrophobic domains, the copolymers present more ordered and distinct phase separated morphologies. These results indicate that the incorporation of DBF and DBT units sharpens the phase boundaries and improves the alignment of the hydrophilic domains, thereby facilitating the formation of continuous ion‐conducting pathways.

Thermal stability was evaluated by thermogravimetric analysis (TGA) and derivative thermogravimetric (DTG) analysis under a nitrogen atmosphere (Figure S16). A small weight loss below 250°C is attributed to residual solvent and absorbed moisture [43]. The PHT membrane exhibits a major weight loss between 300 and 420°C, corresponding to the decomposition of the side chains. In comparison, PHf begins to degrade at around 200°C, indicating a relatively lower thermal stability of the DBF‐based backbone. A second weight loss above 400°C is assigned to the degradation of the aromatic polymer backbone [15, 37]. Quaternized membranes show slightly reduced thermal stability compared to their pristine counterparts. However, all membranes remain thermally stable well above the operating temperature range of AEMWE systems (60–80°C), satisfying any practical requirements.

**FIGURE 3:**
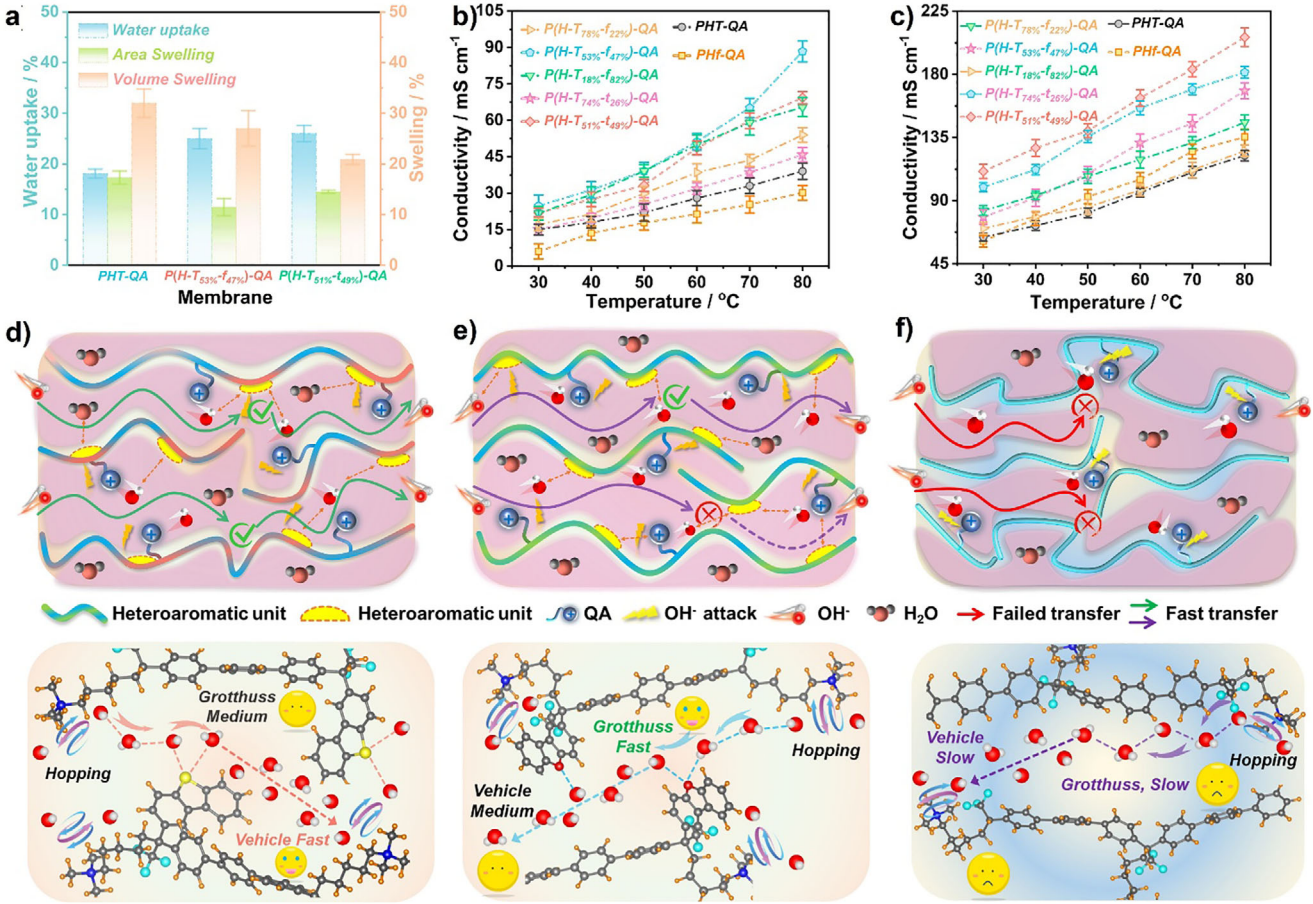
a) Water absorption and swelling, b) Br^−^ and c) OH^−^ ion conductivity of the different AEMs; Schematic diagram illustrating the ion transport process of d) P(H‐T_x_‐t_y_), e) P(H‐T_x_‐f_y_), and f) PHT.

Ion exchange capacity (IEC) data determined by Mohr titration are summarized in Table S2. Theoretical IEC values are calculated from the copolymer compositions. PHT‐QA exhibits the lowest theoretical IEC (1.93 mmol g^−1^), while DBF‐ and DBT‐based copolymers show increased IEC values with increasing contents of the π‐conjugated units. The experimentally measured IEC values are slightly lower than theoretical predictions, likely due to incomplete ion exchange during measurement and incomplete quaternization of the pendant tertiary amine groups, which is also consistent with the grafting degree analysis discussed above. Water uptake (WU) generally increases with IEC; however, it is also strongly influenced by the polymer structure. For instance, PHT‐QA has a relatively high IEC but a low water uptake (18.1%), whereas PHf‐QA reaches 33.8% (Figure 3a), which can be attributed to the increased polarity of the furan units and the larger free volume introduced by the DBF segments. Excessive water uptake, however, can dilute charge carriers and induce excessive swelling, which negatively affects ion transport [27, 31]. Consistently, PHf‐QA exhibits the highest swelling ratio (44.4%), while PHT‐QA shows moderate swelling (32.3%) despite its low water uptake, indicating an expansion of constrained ionic domains upon hydration.

Mechanical properties were evaluated by tensile testing (Figure S17a). All membranes exhibit predominantly elastic behavior. It is well established that polymer molecular weight plays an important role in determining mechanical properties because sufficiently high molecular weights promote chain entanglement and enhance mechanical integrity. Gel permeation high performance liquid chromatography (GPC) analysis confirms that all three representative polymers possess comparable molecular weights (*M*_w_ = 208–241 kDa) and narrow molecular‐weight distributions (PDI = 1.06–1.12; Table S3). Therefore, molecular weight differences are unlikely to be the dominant factor governing the observed mechanical behavior. Instead, the mechanical properties are primarily dictated by the combined effects of water uptake and chain segment structure. PHT‐QA shows a tensile strength of 44.6 MPa, while P(H‐T_78%_‐f_22%_)‐QA achieves a favorable balance of strength (46.1 MPa) and elongation at break (17.1%). Increasing the content of π‐conjugated segments leads to a reduction in both strength and flexibility, as observed for P(H‐T_53%_‐f_47%_)‐QA. PHf‐QA exhibits a significant decrease in tensile strength (22.3 MPa). This reduction is mainly attributed to its high water uptake, which plasticizes the polymer matrix and weakens intermolecular interactions. In addition, the twisted DBF‐containing segments may increase the susceptibility of the hydrated membrane to local stress concentration during deformation. This result highlights the importance of balancing hydration and mechanical robustness in AEM design. Interestingly, the DBT‐based membrane P(H‐T_51%_‐t_49%_)‐QA exhibits a moderate tensile strength (37.4 MPa) and an elongation at break of approximately 20%, demonstrating an attractive balance between strength and ductility. Its water uptake lies between those of P(H‐T_53%_‐f_47%_)‐QA and PHT‐QA, providing sufficient plasticization to enhance chain mobility while preserving membrane integrity. Moreover, the sulfur atom of the DBT units possesses a larger atomic radius and higher polarizability than the oxygen atoms of the DBF units, which may contribute to increased conformational adaptability of the polymer backbone. Such structural characteristics may facilitate stress relaxation and energy dissipation during deformation, thereby improving membrane ductility. Overall, these results demonstrate that the incorporation of π‐conjugated heteroaromatic units and flexible side chains enables a precise regulation of microphase separation, water management, and mechanical properties, providing a synergistic pathway toward high‐performance AEMs for alkaline water electrolysis.

### 2.3 Ion Conduction and Mechanism

Ionic conductivity plays a decisive role in determining the electrochemical performance of AEMWEs. All membranes exhibit increased conductivity with rising temperature, consistent with the expected thermally activated ion transport [12, 38]. In the Br^−^ form (Figure 3b), PHT‐QA shows a low conductivity of 39 mS cm^−1^ at 80°C, which can be attributed to its limited ion mobility and lack of well‐defined transport pathways. The incorporation of DBF or DBT units significantly enhances conductivity, reaching a maximum for P(H‐T_53%_‐f_47%_)‐QA at 88 mS cm^−1^. However, a too high DBF content leads to a decline in conductivity, as observed for PHf‐QA (30 mS cm^−1^), due to excessive water uptake and dilution of charge carriers. As expected, the hydroxide conductivity (Figure 3c), which is more relevant for AEMWE operation, is substantially higher than Br^−^ conductivity owing to the faster diffusion of OH^−^ in aqueous environments [31]. PHf‐QA (135 mS cm^−1^) slightly outperforms PHT‐QA (122 mS cm^−1^), indicating that the furan units facilitate hydroxide transport through hydrogen‐bond assisted pathways. The DBF‐based copolymers achieve their highest conductivity with P(H‐T_53%_‐f_47%_)‐QA (181 mS cm^−1^). Notably, the DBT‐based membranes exhibit superior performance overall, with P(H‐T_51%_‐t_49%_)‐QA reaching 206 mS cm^−1^. This enhancement is attributed to the formation of well‐connected ion transport channels and a favorable microphase separation (Figure 3d).

**FIGURE 4:**
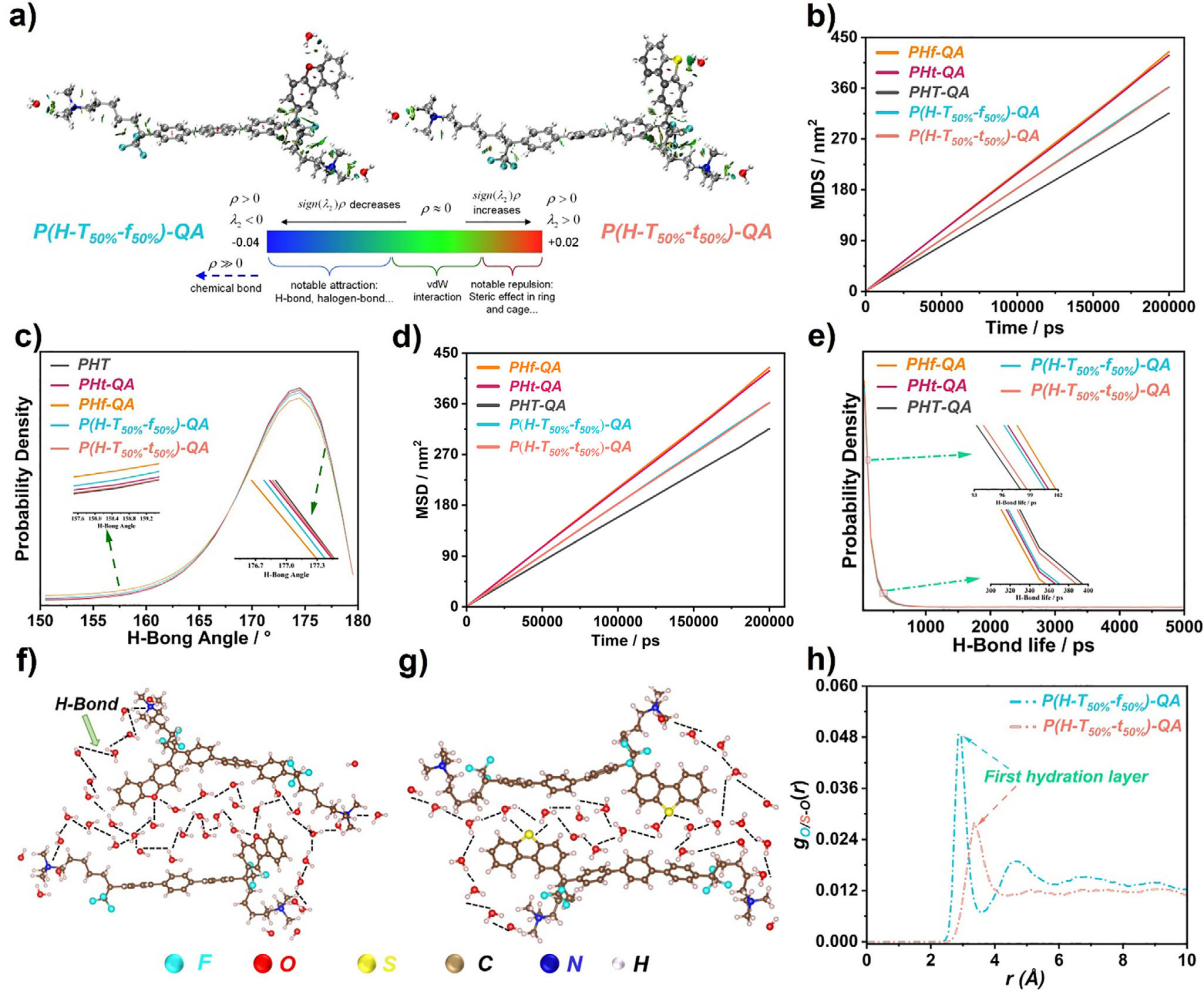
a) Interaction region indicator (IRI), b) mean square displacement (MSD) of OH^−^, c) probability distribution of H‐bond angles in various AEMs, d) statistics of the number of hydrogen bonding during 200 000 ps of various AEMs, e) hydrogen bonding life‐time, f, g) H‐bond network scheme, h) RDF of O/S and H_2_O in P(H‐T_50%_‐f_50%_)‐QA and P(H‐T_50%_‐t_50%_)‐QA.

To gain a molecular‐level insight into the ion transport mechanism, molecular dynamics simulations were performed. An interaction region indicator analysis (Figure 4a) confirms the presence of strong noncovalent interactions in the copolymer systems. The mean square displacement (MSD) profiles of OH^−^ ions exhibit a linear increase with simulation time (Figure 4b), indicating normal diffusive behavior and enabling the calculation of apparent diffusion coefficients. Although MSD analysis alone cannot distinguish between the contributions of vehicular diffusion and site‐hopping transport, the calculated diffusion coefficients provide a quantitative comparison of OH^−^ mobility among the different membranes. In PHT‐QA, the discontinuous distribution of ionic sites results in inefficient long‐range ion transport. In contrast, increasing DBF or DBT content enhances the density of accessible hopping sites, leading to higher OH^−^ diffusion coefficients and improved conductivity (Figure 3d‐f) [36].

Hydrogen bonding network characteristics are intimately linked to ion transport dynamics, as they govern the local chemical environment experienced by the diffusing hydroxide ions. Analysis of hydrogen bonding angle distributions (Figure 4c) reveals that DBF‐ and DBT‐containing copolymers exhibit a higher proportion of small angle hydrogen bonds (<167°) compared to PHT‐QA, indicating denser and more flexible hydrogen bonding networks. While classical MD simulations cannot directly capture the bond breaking and bond forming events inherent to a Grotthuss‐type hopping mechanism, the calculated hydrogen bonding lifetime and density serve as quantitative descriptors of the network's structural dynamics, which are expected to heavily influence the dominant ion transport mode. An optimal balance between hydrogen bonding lifetime and concentration is recognized as crucial for efficient ion transport: excessively long lifetimes hinder network reorganization, while excessively short lifetimes destabilize the pathway. As illustrated in Figure 4d,e, both the oxygen in DBF and the sulfur in DBT can act as additional hydrogen bonding acceptors, facilitating the formation of a continuous hydrogen bonding network. PHT‐QA exhibits the longest hydrogen bonding lifetime (125.97 ps) but the lowest number of hydrogen bonds, suggesting sluggish structural reorganization that correlates with its lower conductivity. In contrast, PHf‐QA displays a shorter lifetime (111.12 ps) coupled with a higher hydrogen bonding count, reflecting a more interconnected hydrogen bonding network. A consistent trend is observed in the copolymers: DBF‐containing membranes maintain a higher hydrogen bonding density alongside shorter lifetimes (Figure 4f,g). We propose that this combination of dense, yet rapidly exchanging, hydrogen bonding network may provide a structural environment that is particularly conducive to a Grotthuss‐like contribution, complementing the vehicular transport enabled by the simultaneously increased free volume. FTIR analysis further supports these findings (Figure S18). It has previously been reported that lower O─H stretching frequencies indicate stronger hydrogen bonding interactions [20, 36, 44]. PHf‐QA exhibits the lowest stretching frequency at 3380 cm^−1^, suggesting restricted water mobility and strong hydrogen bonding interactions. DBT‐based membranes show slightly higher frequencies than their DBF counterparts, indicating less dense but more extended hydrogen bonding networks. PHT‐QA exhibits the highest frequency at 3394 cm^−1^, consistent with its weaker hydrogen bonding interactions as revealed by the MD simulations.

A radial distribution function (RDF) analysis was conducted to probe the local hydration structure around heteroatoms (Figure 4h) [42, 45, 46]. The g_S‐O_(r) and g_O‐O_(r) profiles quantify the spatial probability distributions of water molecules or OH^−^ ions near sulfur and oxygen atoms, respectively. The first RDF peak represents the primary hydration shell of water molecules around heteroatoms, defining the effective range of hydrogen bonding interactions [44, 47]. The RDF profiles reveal that the sulfur atoms in DBT form extended hydration structures over distances of 3.05–3.95 Å, while oxygen atoms in DBF form shorter and tighter hydration shells (2.60–3.55 Å). These contrasting hydration patterns indicate that the two π‐conjugated heterocycles bring out fundamentally different local chemical environments. DBT promotes a more spatially extended and loosely bound water network, whereas DBF favors a compact and strongly coordinated hydration structure. The compact hydration around DBF is consistent with a dense hydrogen bonding network, which, in combination with the increased free volume, may create an environment conducive to both Grotthuss‐like hopping and vehicular diffusion. Conversely, the extended hydration around DBT, coupled with its more pronounced phase separation and larger free volume, appears to favor transport that is more strongly vehicle‐dominated. It should be noted that the classical MD simulations employed here cannot explicitly resolve the bond‐breaking and bond‐forming events characteristic of the Grotthuss mechanism; the proposed strategy is therefore a structure–property hypothesis derived from indirect descriptors, rather than a definitive mechanistic proof. The superior overall hydroxide conductivity observed in DBT‐containing membranes likely arises from a synergistic contribution of efficient phase separation and the formation of continuous, long range ion transport pathways. Collectively, the experimental findings demonstrate that π‐conjugated heterocycles synergistically modulate both the electronic structure of the framework and the resultant microphase morphology, two interdependent factors that critically govern ion transport behavior. Crucially, it is the dynamic interplay between hydrogen bonding networks and phase separated microstructure, not a simplistic dichotomy based on Grotthuss‐ or vehicle‐type conduction mechanisms, that dictates the anion transport performance of these AEMs.

### 2.4 Alkaline Stability

**FIGURE 5:**
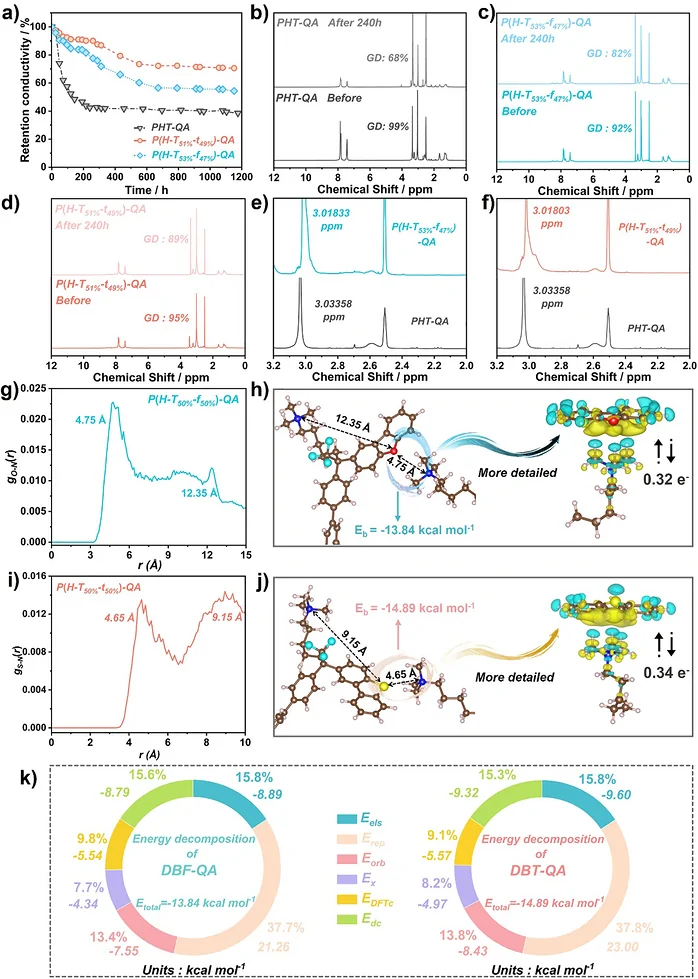
a) Long‐term conductivity retention in 1 m KOH at 80 ^o^C; b–d) ^1^H NMR spectra before and after a 240‐h alkaline stability test of the PHT‐QA, P(H‐T_53%_‐f_47%_)‐QA, and P(H‐T_51%_‐t_49%_)‐QA membranes; e, f) Expanded ^1^H NMR spectra of PTP‐QA, P(H‐T_53%_‐f_47%_)‐QA, P(H‐T_51%_‐t_49%_)‐QA; g, i) RDF of S and N in P(H‐T_50%_‐f_50%_)‐QA, and O and N in P(H‐T_50%_‐t_50%_)‐QA; h, j) ionic dipole interactions, DFT differential charge densities and k) interaction energies of the DBF and DBT units, respectively, with the QA cation.

The chemical stability of cationic functional groups under alkaline conditions remains a key limitation for long‐term AEMWE operation [11, 16, 29]. The durability of the membranes was evaluated by monitoring the conductivity retention in 1 m KOH at 80°C (Figure 5a). PHT‐QA exhibits rapid degradation, retaining only 44% of its initial conductivity after 240 h. In contrast, the copolymer membranes show significantly improved stability, with P(H‐T_51%_‐t_49%_)‐QA and P(H‐T_53%_‐f_47%_)‐QA retaining 91% and 84%, respectively, of their initial conductivity. ^1^H NMR spectra of the aged membranes (Figure 5b–d) reveal a gradual decrease in grafting degree (GD) is observed. For P(H‐T_51%_‐t_49%_)‐QA, the GD decreases from 95% to 89%, while the decrease for P(H‐T_53%_‐f_47%_)‐QA is from 92% to 82%. In contrast, PHT‐QA shows a pronounced GD decrease from 99% to 68%, accompanied by the appearance of a signal from a degradation product at 4.05 ppm. After prolonged exposure, the conductivity retention stabilizes at 71% for P(H‐T_51%_‐t_49%_)‐QA, 54% for P(H‐T_53%_‐f_47%_)‐QA, and only 39% for PHT‐QA, confirming the superior alkaline stability of the copolymer membranes. To further elucidate the alkaline degradation pathways, accelerated aging tests were conducted in 5 m KOH solution at 80°C for 240 h. After aging, ^1^H NMR spectra revealed QA retention ratios of 53%, 33%, and 17% for P(H‐T_51%_‐t_49%_)‐QA, P(H‐T_53%_‐f_47%_)‐QA, and PHT‐QA, respectively (Figure S19a). In addition, characteristic signals associated with quaternary ammonium degradation were observed, including vinyl proton signals originating from E2 elimination (≈2.0 ppm), ─N(CH_3_)_2_ end groups generated through α‐carbon S_N_2 degradation (≈2.5 ppm), and ─CH_2_OH termini formed through β‐carbon S_N_2 degradation (≈3.5 ppm) (Figure S19b). These results further confirm that the incorporation of DBT units effectively suppresses alkaline degradation. Meanwhile, tensile measurements were conducted after alkaline stability testing in 1 m KOH at 80°C to evaluate mechanical stability (Figure S17b). Both copolymer membranes exhibit a similar trend, characterized by a moderate decrease in tensile strength accompanied by a noticeable increase in elongation at break. Specifically, P(H‐T_53%_‐f_47%_)‐QA shows an increase in elongation from 8.2% to 14.9%, while the tensile strength decreases from 35.1 to 28.9 MPa. Similarly, P(H‐T_51%_‐t_49%_)‐QA exhibits an increase in elongation from 19.3% to 25.1%, followed by a decrease in tensile strength from 37.4 to 30.1 MPa. The increased ductility after alkaline aging is likely associated, at least in part, with the partial loss of QA groups, which reduces steric constraints and increases segmental mobility within the hydrated polymer matrix. Meanwhile, the relatively small decrease in tensile strength suggests that severe degradation of the polymer framework did not occur under the testing conditions. Notably, P(H‐T_51%_‐t_49%_)‐QA retains the highest post‐aging elongation at break (25.1%) together with a strength retention of 80.4%, indicating a favorable balance between flexibility and mechanical robustness after prolonged alkaline exposure. These results provide experimental evidence that the DBT‐containing membrane maintains superior structural resilience under alkaline operating conditions.

Molecular simulations were performed to clarify the origin of the enhanced stability of the copolymer AEMs. Radial distribution function analysis (Figure 5g,i) reveals distinct spatial correlations between the heteroatoms (S or O) and the nitrogen atoms of the QA groups. In DBT‐based membrane of P(H‐T_50%_‐t_50%_)‐QA, a strong S···N interaction is observed, with g_S‐N_(r) characteristic peaks at 4.65 and 9.35 Å, corresponding to intermolecular and intramolecular interactions, respectively. The shorter distance indicates strong ion‐dipole coupling between DBT segments and the QA groups, which stabilizes the cationic sites against nucleophilic attack [35, 36]. In contrast, DBF‐based membranes exhibit slightly longer O···N distances (approximately 4.75 Å), suggesting weaker ion–dipole interactions despite the higher electronegativity of oxygen. DFT calculations further support this conclusion. As shown in Figure 5h,j, the interaction energy between DBT and QA groups (‐14.89 kcal mol^−1^) is stronger than that for DBF (‐13.84 kcal mol^−1^), accompanied by a greater electron transfer (0.34 e vs 0.32 e). Energy decomposition analysis (Figure 5k) indicates that DBT benefits from stronger electrostatic and orbital interactions (*E*_els_ = ‐9.60 kcal mol^−1^ and *E*_orb_ = ‐8.43 kcal mol^−1^, respectively), which can be attributed to the larger atomic radius and more polarizable electron cloud of the sulfur atom [48]. These electronic interactions are also reflected in the ^1^H NMR chemical shifts. As depicted in Figure 5e,f, the QA methyl signal shifts from 3.0336 ppm in PHT‐QA to 3.0180 ppm in P(H‐T_51%_‐t_49%_)‐QA, indicating increased electron density around the cationic center. A smaller shift (3.0183 ppm) is observed for the P(H‐T_53%_‐f_47%_)‐QA membrane. Together, these results demonstrate that the stronger ion‐dipole interactions in DBT‐containing copolymers effectively stabilize the QA groups, leading to enhanced alkaline durability.

### 2.5 AEMWE Performance and Durability

**FIGURE 6:**
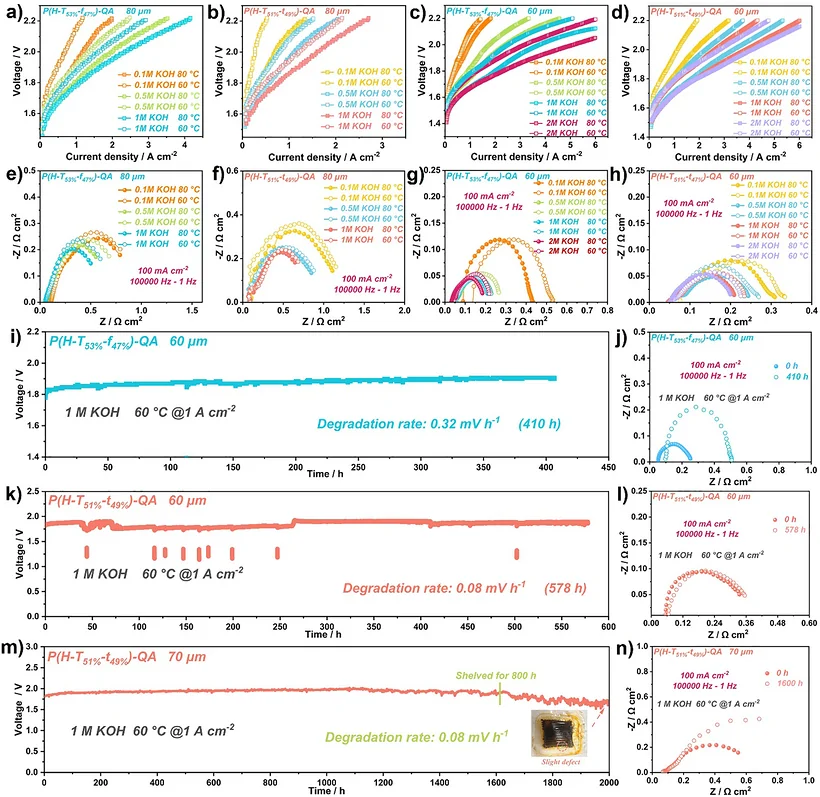
a–d) Polarization curves and e–h) EIS spectra of water electrolysis cells assembled with 60‐ and 80‐µm thick P(H‐T_53%_‐f_47%_)‐QA and P(H‐T_51%_‐t_49%_)‐QA membranes under various operating conditions; i, k, m) durability performance of 60 and 70 µm thick P(H‐T_53%_‐f_47%_)‐QA, P(H‐T_51%_‐t_49%_)‐QA membranes operating with 1 m KOH solution at a current density of 1 A cm^−2^ at 60°C; j, l, n) EIS spectra before and after the durability tests.

After the comprehensive physicochemical characterization, P(H‐T_53%_‐f_47%_)‐QA and P(H‐T_51%_‐t_49%_)‐QA were, based on their overall favorable properties, selected for AEMWE evaluation using non‐precious Ni─Fe catalysts. Building upon the structure‐property relationships discussed above, we sought to understand how the distinct hydration environments and morphologies, compact and hydrogen‐bond rich for DBF, extended and phase separated for DBT, translate into electrolysis performance under realistic operating conditions. The electrolysis performance was first evaluated at varying KOH concentrations and membrane thicknesses. Increasing temperature and electrolyte concentration enhances ion mobility and reduces ohmic resistance [14, 31, 49, 50], resulting in improved cell performance (Figure 6a,b). For an 80 µm thick P(H‐T_53%_‐f_47%_)‐QA membrane, increasing the KOH concentration from 0.1 to 1 m raises the current density from 1.2 to 2.3 A cm^−2^ at 2 V. This is accompanied by a 41.6% decrease in membrane resistance (0.089 to 0.051 Ω), as determined by electrochemical impedance spectroscopy (Figure 6e). In comparison, P(H‐T_51%_‐t_49%_)‐QA shows a smaller increase in current density, from 0.7 to 1.6 A cm^−2^, and a 30.3% reduction in resistance (0.099 to 0.069 Ω) under identical conditions (Figure 6f). The stronger sensitivity of DBF‐based membranes to KOH concentration is consistent with their more compact hydration structure, where the availability of charge carriers in the electrolyte more directly influences the local ion population within the transport channels.

Reducing membrane thickness significantly improves performance by shortening ion transport pathways [51]. For P(H‐T_53%_‐f_47%_)‐QA, decreasing the thickness from 80 to 60 µm increases the current density by 63.0% (2.3 to 3.75 A cm^−2^ at 2 V and 1 m KOH) and reduced resistance by 29.8% (0.057 to 0.040 Ω) (Figure 6c,g). In contrast, P(H‐T_51%_‐t_49%_)‐QA exhibits a more pronounced improvement, with the current density increasing by 108.1% (1.85 to 3.85 A cm^−2^) and the resistance decreasing by 34.8% (Figure 6d,h). This strong thickness dependence is further confirmed by additional tests using 70 and 55 µm P(H‐T_51%_‐t_49%_)‐QA membranes, respectively, as shown in Figure S20. A reduction from 80 to 70 µm yields an 89.2% increase in current density (1.85 to 3.5 A cm^−2^ at 2 V), surpassing the 63.0% improvement for P(H‐T_53%_‐f_47%_)‐QA after decreasing the thickness from 80 to 60 µm. Meanwhile, a further reduction from 60 to 55 µm for P(H‐T_51%_‐t_49%_)‐QA results in a performance gain (current density at 2 V: 3.85 to 4.1 A cm^−2^) comparable to doubling the KOH concentration (from 1 to 2 m) for P(H‐T_51%_‐t_49%_)‐QA (60 µm, from 3.85 to 4.12 A cm^−2^, Figure 6d). The pronounced thickness sensitivity observed in DBT‐based membranes might result from their larger free volume and more extended phase separated morphology, where ion transport may rely more heavily on long range diffusional pathways that scale directly with membrane thickness.

Furthermore, increasing KOH concentration from 1 to 2 m results in a substantial enhancement for the DBF‐based membrane, while only a marginal improvement is observed for the DBT‐based system. For example, the current density of P(H‐T_53%_‐f_47%_)‐QA (60 µm) increases from 3.75 to 5.12 A cm^−2^, accompanied by a 27.5% reduction in resistance (from 0.040 to 0.029 Ω) as depicted in Figure 6c,g, whereas P(H‐T_51%_‐t_49%_)‐QA (60 µm) shows only a minor increase from 3.85 to 4.12 A cm^−2^ along with a 6.7% reduction of resistance (Figure 6d,h). These contrasting responses to electrolyte concentration and membrane thickness can be rationalized by the distinct microstructural environments in the two membranes. The compact, hydrogen‐bond rich hydration structure of DBF‐based membranes renders their ion transport more sensitive to the availability of hydroxide ions in the electrolyte, whereas the extended, phase separated morphology of DBT‐based membranes leads to transport that is more dominated by long range diffusional pathways, thereby responding more strongly to changes in membrane thickness. And these structural characteristics give rise to complementary transport behaviors. The dense hydration networks around DBF units facilitate efficient local ion migration, particularly at elevated hydroxide concentrations, while the continuous phase separated channels in DBT‐based systems enable effective long‐range transport, which can be substantially enhanced by reducing the membrane thickness. This complementarity provides a practical strategy for tuning membrane performance under different operating conditions.

Long‐term durability was evaluated under practical AEMWE operating conditions (1 m KOH, 60°C, 1 A cm^−2^) [18, 31, 52]. Using 60‐µm thick membranes, the cell assembled with P(H‐T_53%_‐f_47%_)‐QA exhibited a voltage increase from 1.778 to 1.907 V after 410 h of operation, corresponding to a degradation rate of 0.32 mV h^−1^ (Figure 6i). The Nyquist plots (Figure 6j) reveal increases in both the high frequency resistance (HFR) and polarization resistance after the durability testing. As shown in Figure S21, the EIS spectra were fitted using an equivalent circuit consisting of an ohmic resistance (*R*_m_) in series with a parallel combination of a constant phase element (*C*_d_) and polarization resistance (*R*_p_). The *R*_m_ value mainly reflects membrane related ionic resistance, whereas *R*_p_ represents the overall polarization contribution from electrode kinetics, catalyst layer ionic transport, and electrode‐membrane interfacial processes. Therefore, the increase in *R*_m_ is primarily associated with reduced hydroxide conductivity caused by membrane degradation, while the increase in *R*_p_ may originate from combined changes in electrode polarization and interfacial processes. In contrast, the P(H‐T_51%_‐t_49%_)‐QA (60 µm) based cell exhibited a much lower degradation rate, 0.08 mV h^−1^ over 578 h (Figure 6k), accompanied by only slight increases in *R*_m_ and *R*_p_ (Figure 6l and Table S4), demonstrating improved membrane durability and stable electrode operation. These results demonstrate the superior operational stability of the DBT‐based membranes. Extended durability testing further highlights the robustness of P(H‐T_51%_‐t_49%_)‐QA. Using a 70‐µm thick membrane, stable operation was maintained for over 1600 h with minimal voltage increase and negligible resistance change (Figure 6m), indicating sustained ion‐transport performance. However, as depicted in Figure 6n, after a 1600‐h operation, the increase in *R*_p_ becomes considerably more pronounced, whereas HFR remains relatively stable. This behavior suggests that electrode or interfacial degradation becomes a contributor to performance decay. In addition, the increase of *R*_p_ may also be attributed to a possible membrane leakage. When leakage pathways develop during long‐term operation, a portion of the applied current may be leakage current and thus does not participate in the desired electrochemical reactions. Hence, a fraction of the applied current may bypass the desired electrochemical reactions. According to the Butler‐Volmer relationship $\left(R_{ct}=\frac{RT}{\alpha Fi}\;\right)$, the charge‐transfer resistance (R_ct_) is inversely proportional to the exchange current density and then leads to an increase of *R*_p_. After a standby period of 800 h, the system resumed operation with voltage drop and fluctuation and continued for an additional 422 h before mechanical failure occurred after a total operating time of 2022 h (Figure 6m). Post‐test ^1^H NMR analysis reveals that approximately 77% of the grafted cationic groups remained after 2022 h of operation (Figure S22), corresponding to a loss of about 18% of the QA groups. This degree of degradation is noticeably lower than that observed in the ex situ alkaline stability tests (Figure 5a), suggesting that the continuously hydrated operating environment and electrolyte circulation may partially mitigate chemical degradation during practical AEMWE operation. These results highlight the importance of evaluating membrane stability under realistic operating conditions. Overall, the DBT‐based membrane achieves an optimal balance between conductivity, mechanical robustness, and alkaline stability, enabling long‐term and reliable operation in AEMWE systems. This study demonstrates that rational tuning of the π‐conjugated heterocycle structures and copolymer composition offers an effective means to manipulate the hydration structure, microphase morphology, and ultimately the ion transport, and provides an effective pathway toward high‐performance and durable AEMs.

## 3 Conclusion

In this work, we developed a series of structurally tunable AEMs by integrating π‐conjugated heterocyclic units with long cationic alkyl side chains. The incorporation of oxygen‐ and sulfur‐containing heterocyclic building blocks enables simultaneous regulation of hydrogen bonding interactions and microphase separation, leading to significantly enhanced hydroxide‐ion transport. More importantly, this study reveals that subtle differences in heteroatom chemistry, including electronegativity, atomic radius, and polarizability, can generate distinct hydration environments and ion‐transport characteristics. Specifically, DBF units promote the formation of dense hydrogen bonding networks, whereas DBT units facilitate stronger ion‐dipole interactions and more pronounced microphase separation. We propose that these structural differences give rise to distinct transport characteristics, thereby enabling a favorable balance between hydroxide conductivity and alkaline stability. Consequently, the optimized membrane achievesZ hydroxide conductivities as high as 206 mS cm^−1^ together with markedly improved stability. When applied in AEMWE devices, these membranes deliver high current densities and excellent operational stability. In particular, the DBT‐based membrane exhibits stable operation for over 1600 h, demonstrating strong resistance to both chemical and mechanical degradation. Overall, this work establishes a clear relationship between chemical structure, ion transport and device performance, and provides a general molecular design strategy based on the incorporation of π‐conjugated heterocycles units into copolymer backbones for the development of durable, high‐performing AEMs. The insights presented here offer a practical route toward durable high‐performing membranes for alkaline electrochemical energy conversion systems.

## Author Contributions

**Qian Wang**: conceptualization, investigation, methodology, visualization, and writing – original draft. **Yang Wu**: software. **Patric Jannasch**: conceptualization, resources, writing – original draft, and writing – review and editing. **Si Chen**: formal analysis. **Yunpeng Guo**: software. **Jingshuai Yang**: conceptualization, funding acquisition, project administration, resources, supervision, writing – original draft, and writing – review and editing. **Yun Zhao**: funding acquisition, supervision, writing – review and editing, and writing – original draft. **Wenzhe Zhao**: formal analysis, investigation, visualization, and writing – original draft. **Riyang Huang**: data curation, investigation, validation, and writing – original draft.

## Conflicts of Interest

The authors declare no conflicts of interest.

## Supporting information




**Supporting File**: advs77491‐sup‐0001‐SuppMat.pdf.

## Data Availability

The data that support the findings of this study are available from the corresponding author upon reasonable request.
