# Supplementary material for: Tailoring Structure–Property Relationships in π‐Conjugated Heterocyclic Poly(arylene alkylene) Anion‐Exchange Membranes for High‐Performance Water Electrolysis
Source: Adv Sci (Weinh). 2026 Aug 31:e77491. Online ahead of print. doi: 10.1002/advs.77491 (PMC13530020; doi:10.1002/advs.77491)
Supplement: Supplementary file 1 [file ADVS-9999-e77491-s001.pdf]

Supporting Information

Tailoring Structure-Property Relationships in π-Conjugated Heterocyclic Poly(arylene alkylene) Anion-Exchange Membranes for High-Performance Water Electrolysis

Qian Wang[a,1], Riyang Huang[b,1], Wenzhe Zhao[a,1], Si Chen[c], Yunpeng Guo[a], Yang Wu[d], Yun Zhao* [b], Patric Jannasch* [c], Jingshuai Yang* [a, c]

[a] Q. Wang, W. Zhao, Y. Guo, J. Yang
Department of Chemistry, College of Sciences
Northeastern University
Shenyang 110819, China
E-mail: yjs@mail.neu.edu.cn

[b] R. Huang, Y. Zhao
Fuel Cell System and Engineering Laboratory, Key Laboratory of Fuel Cell & Hybrid Power Sources
Dalian Institute of Chemical Physics, Chinese Academy of Sciences
Dalian, 115623, China
E-mail: yunzhao@dicp.ac.cn

[c] S. Chen, P. Jannasch
Polymer & Materials Chemistry, Department of Chemistry
Lund University
P.O. Box 124, SE-221 00 Lund, Sweden
E-mail: patric.jannasch@chem.lu.se

[d] Y. Wu
College of Chemistry
Liaoning University
Shenyang 110036, Liaoning, China

[1] These authors contributed equally to this work.

Table of contents

Experimental 2
Characterizations 2
DFT calculations 3
Molecular dynamics simulations 3
Supplementary Figures and Table 4
References 17

Experimental

Materials

Dibenzofuran (DBF), dibenzothiophene (DBT), *p*-terphenyl (TP), 7-bromo-1,1,1-trifluoro-2-heptanone, trimethylamine (25% in water), trifluoromethanesulfonic acid (TFSA) were supplied by Adamas Reagent Ltd. Other chemicals, including dichloromethane (DCM), sodium bicarbonate ($NaHCO_3$), potassium hydroxide (KOH), N,N-dimethylformamide (DMF), N,N-dimethylacetamide (DMAc), dimethyl sulfoxide (DMSO) and N-methylpyrrolidone (NMP) were obtained from Sinopharm Chemical Reagent Co. Ltd. All reagents were used without further purification.

Synthesis of related polymers and copolymers

The polymers were synthesized via Friedel-Crafts polyhydroxyalkylations involving 7-bromo-1,1,1-trifluoro-2-heptanone and various aromatic compounds (including TP, DBF and DBT) under superacid catalysis, as depicted in Figure S1. As a typical example, the synthesis of PHT from *p*-terphenyl and 7-bromo-1,1,1-trifluoro-2-heptanone. In this process, *p*-terphenyl (1.00 g, 4.34 mmol) was uniformly dispersed in dichloromethane (DCM) in a 100 mL round-bottom flask. Subsequently, 7-bromo-1,1,1-trifluoro-2-heptanone (1.18 g, 4.78 mmol) was added dropwise to the solution. After thorough stirring, trifluoromethanesulfonic acid (TFSA, 3.85 mL) was introduced after ice-bath cooling to initiate the polymerization reaction. The ice bath was promptly removed upon TFSA addition. Following a 4 h reaction at room temperature, the reaction mixture exhibited a pink color and reached a high viscosity. The mixture was then poured into an aqueous sodium bicarbonate ($NaHCO_3$) solution for precipitation. The resulting fiber-like polymer was chopped-up and repeatedly washed with deionized water to remove residual alkaline substances. It was subsequently dried in an oven at 80 °C, yielding the white polymer PHT.

The synthesis of the copolymers deviates slightly from the aforementioned procedure. Taking poly(trphenyl dibenzofuran 7-bromo-1,1,1-trifluoro-2-heptanone) (P(H-$T_{53\%}$-$f_{47\%}$)) as an example. After adding TFSA under ice bath conditions and stirring at room temperature for 45 min, the reaction system turned brown and highly viscous. This indicates that DBF exhibits a higher reactivity compared to the other monomers. The electron density distribution of the aromatic monomers plays a decisive role in governing the polymerization kinetics. The double descriptor (Δf), derived from conceptual density functional theory (CDFT), is a reliable indicator for evaluating electrophilic reactivity.[1] As shown in Figure S2, the double descriptor value of dibenzothiophene (DBT) is -0.0329, significantly higher than those of DBF (-0.0115) and *p*-terphenyl (-0.0021). Higher reactivity accelerates the reaction rate. Due to the very rapid and uncontrollable polymerization process, the PHT homopolymer was not prepared in this study. Upon completion of the copolymerization, an additional 10 mL of NMP solution was added to facilitate stirring and terminate the reaction. The brown solution was then precipitated in an aqueous $NaHCO_3$ solution. The synthesis procedures for all copolymers were the same as for P(H-$T_{53\%}$-$f_{47\%}$), and they were named P(H-T_x-f_y) or P(H-T_x-t_y) depending on the types of monomers involved, where x denotes the proportion of *p*-terphenyl in the copolymer backbone, and y represents the proportion of DBF or DBT molecules in the main chain composition. Both x and y were determined by the ^{1}H NMR results.

Preparation of QA-grafted membranes

An aqueous solution of trimethylamine was employed as the quaternization reagent to displace the terminal bromine atoms of the side chains by nucleophilic substitution reactions, thereby attaching the QA groups onto the polymer chain. Owing to the reduced steric hindrance away from the main chain, the quaternization process does not require any catalysts or heating. Taking the quaternization of PHf as an example, 0.5 g of the polymer was dispersed in 15 mL aqueous trimethylamine solution. Continuous stirring at 50 °C for 48 h facilitated the quaternization reaction, during which the polymer gradually dissolved in the trimethylamine solution. Subsequently, the reactor was transferred to an oven at 80 °C for 24 h to evaporate and remove excess trimethylamine solution. The filtrated solution was poured on an ultra-flat Petri dish and placed in an oven at 80 °C to form a membrane. The final membrane was washed with deionized water for 12 h to remove residual solvent. The resulting membrane was washed with deionized water and dried.

Characterizations

^{1}H NMR spectra were acquired using a BRUKER AVANCE 400 instrument with DMSO-d_6 as the solvent. ATR-FTIR spectra were collected with a Bruker Vertex 70 instrument. X-ray diffraction data were obtained with an Empyrean instrument and X-ray photoelectron spectroscopy (XPS) of the membranes was carried out using a Thermo-Kalpha instrument. Surface microstructures were observed with an SU-8010 instrument, and microphase separation was analyzed with a Dimension Icon atomic force microscope and a JEM2100PLUS transmission electron microscope. Stress-strain data were collected using a CMT2000 electronic universal testing machine. Conductivity measurements were performed by electrochemical impedance spectroscopy (EIS) using a Princeton electrochemical workstation. Equation S1 is used for the calculation of grafting degree (m represents any methylene group on the alkane chain).

$$GD(\%) = \frac{2H_{a'}}{9H_m} \qquad (1)$$

Gel permeation chromatography (GPC) analysis was conducted using a LC-2050C LT (Shimadzu) system. Prior to analysis, polymer samples were dissolved under heating with continuous magnetic stirring. The mobile phase consisted of NMP. Measurements were performed at 35 °C and a flow rate of 1 mL min^{-1}.

Water uptake, swelling and IEC

Water uptake and swelling were determined by comparing the weight and dimensions of the membranes before and after water absorption. Each membrane sample was first thoroughly dried and weighed (m_0), and its dimensions (L_0, W_0, D_0) were recorded. It was then immersed in water at 30 °C for a duration of 48 h. Next, the membrane was wiped off with a filter paper before measuring the weight (m_1) and dimensions (L_1, W_1, D_1) in the swollen state. The water uptake (WU%) and volume swelling (VS%) were calculated using Equations S2 and S3:

$$WU\% = \frac{m_1 - m_0}{m_0} \times 100\% \qquad (2)$$

$$VS\% = \frac{L_1 W_1 D_1 - L_0 W_0 D_0}{L_0 W_0 D_0} \times 100\% \qquad (3)$$

The ion exchange capacity (IEC) of the membranes was determined using the Mohr method. The membrane was first soaked in a 0.5 M aqueous KNO_3 solution for 48 h to exchange Cl^- with NO_3^-, followed by the addition of K_2CrO_4 as the indicator. Titration with a 0.1 M aqueous $AgNO_3$ was performed until a brick-red precipitate (Ag_2CrO_4) formed. The IEC value was then calculated using Equation S4, and the hydration number was derived from the water uptake employing Equation S5:

$$IEC = \frac{V_{Ag^+} \times 0.1}{m_0} \qquad (4)$$

$$\lambda = \frac{WU\% \times 1000}{18 \times IEC} \qquad (5)$$

DFT calculations

All quantum chemical calculations in this study were performed within the framework of density functional theory. Geometric optimizations and frequency analyses were conducted using the Gaussian 16 C.01 software package at the B3LYP-D3(BJ)/def2-TZVP level of theory [2], where the D3(BJ) dispersion correction was employed to accurately account for long-range dispersion interactions. To confirm that all optimized structures correspond to true minima on the potential energy surface, vibrational frequency calculations were carried out at the same theoretical level, ensuring the absence of imaginary frequencies. For more accurate electronic structure characterization, single-point energy calculations were subsequently performed using the larger def2-TZVPP basis set, while retaining the optimized geometries. The resulting wave-function data were utilized for further electronic structure analysis. Solvent effects in aqueous solution were modeled using the SMD implicit solvation model within a non-equilibrium solvation framework, with thermodynamic integration applied to effectively describe solute–solvent interactions. Electronic structure analyses, including molecular orbital distributions, electrostatic potential (ESP) maps, condensed Fukui functions (CDD), and electron density difference (EDD) plots, were conducted using Multiwfn_3.8_dev,[3,4] with visualization performed via VMD 1.9.3.

A multilevel conformational search strategy was employed as follows: first, a 100-ps molecular dynamics (MD) simulation of the monomer was carried out at 400 K using the xTB 6.7.0 program based on the GFN-FF force field (time step: 1 fs),[5] during which 2000 conformational snapshots were collected at 50-fs intervals. These initial conformers were then geometrically optimized at the GFN0-xTB level using Molclus 1.12.[6] Redundant structures were eliminated through structural clustering with an RMSD threshold of 0.5 Å implemented in the isostat module. The remaining conformers were further refined using GFN2-xTB optimization in combination with the GBSA implicit solvent model to obtain a preliminary energy ranking.[7] In the final refinement stage, high-precision DFT-based calculations were performed: gas-phase geometry optimization and vibrational frequency analysis were completed at the B3LYP-D3(BJ)/6-31G* level, and thermal corrections to free energy were estimated using the quasi-harmonic approximation. Solvation free energies were computed at the B3LYP/def2-TZVPP level with the SMD solvation model. Finally, the Boltzmann-weighted population distribution at 298.15 K was determined based on the total free energies using the isostat module.

Molecular dynamics simulations

In the molecular dynamics simulations, the initial conformation of the polymer was constructed by Gaussview. To ensure comparability across all systems, while maintaining a consistent total atom number and system size, the degree of polymerization was carefully adjusted according to the polymer structure. Homopolymer chains (PHT-QA, PHf-QA, and PHt-QA) each contain 20 repeating units, corresponding to single-chain molecular weights of 8800, 7560, and 7800 g mol^{-1}, respectively. Copolymer chains (DBF-based and DBT-based systems) each contain 10 repeating units, with molecular weights of 8160 and 8320 g mol^{-1}, respectively. This adjustment accounts for the higher unit mass of copolymer repeating units, which contain both terphenyl and heteroaromatic segments.

Reducing the number of repeating units for copolymers ensures that the resulting single-chain molecular weights remain within a narrow range (7560-8800 g mol^{-1}), enabling a balanced comparison across all systems. Each simulation cell contains 10 polymer chains, 200 OH^- counterions, and 5000 OPC3 water molecules in a cubic periodic box. The atomic charge parameters of the polymer chain were obtained by Open Babel 3.1.1 based on the MMFF94 force field calculation.[8] The atomic charge of OH^- was obtained by fitting the electrostatic potential using the RESP2 method at the B3LYP/6-311G(d,p) level, and its force constant was generated based on the quantum chemical Hessian matrix through the redundant internal coordinate Hessian diagonal element (DRIH) method. Non-key parameters are defined using the GAFF force field, and the topology file was automatically generated through the Sobtop_1.0(dev5) program.[9]

The balancing and annealing process is carried out in the following steps. The system pretreatment follows a strict energy minimization-pre-balancing protocol: Firstly, 5000 steps of energy relaxation were carried out using the conjugate gradient method. Subsequently, 1 ns equilibrium simulation was conducted under the NPT ensemble (T=298.15 K, P=1 atm), and the C-rescale pressure coupling and V-rescale temperature coupling methods were adopted. The relaxation times for both temperature and pressure are set at 0.1 ps. The annealing process adopts a six-stage temperature cycle: 0→300 K (0-200 ps), 300→600 K (200-600 ps), 600→300 K (600-1000 ps), with three cycles. Pressure coupling was carried out using the isotropic Parrinelo-Rahman method, and the isothermal compression ratio was set at 4.5×10-5 bar-1. Finally, a long-range simulation of 300 ns was conducted under the NVT ensemble, with a time step of 1 fs. The electrostatic interaction treatment adopts the smooth particle mesh Ewald (SPME) method, with a truncation radius of 1.1 nm, and the Lorentz-Berthelot combination rule is used to handle the van der Waals interaction. Temperature control is achieved through the V-rescale algorithm (τ=0.1 ps). Trajectory analysis covers radial distribution functions and hydrogen bonds. The network (geometric criterion: d(O⋯H) ≤ 3.5 Å, θ ≥ 150°) and the ion diffusion coefficient (Einstein relation) were all accomplished through the built-in tool of GROMACS 2025.1. The free volume was accomplished using the Multiwfn_3.8_dev program, and the visualization processing was implemented using VMD 1.9.3 and Materials Studio.[10]

It should be noted that the simulated chain lengths (20 repeating units for homopolymers, 10 for copolymers) are shorter than the experimentally determined degree of polymerization (Mn ≈ 190–215 kDa). However, the single-chain molecular weights across all five systems fall within a narrow range (7560-8800 g mol^{-1}). All comparative analyses were performed under identical simulation conditions, ensuring the reliability of the observed trends between DBF- and DBT-based systems.

Supplementary Figures and Table

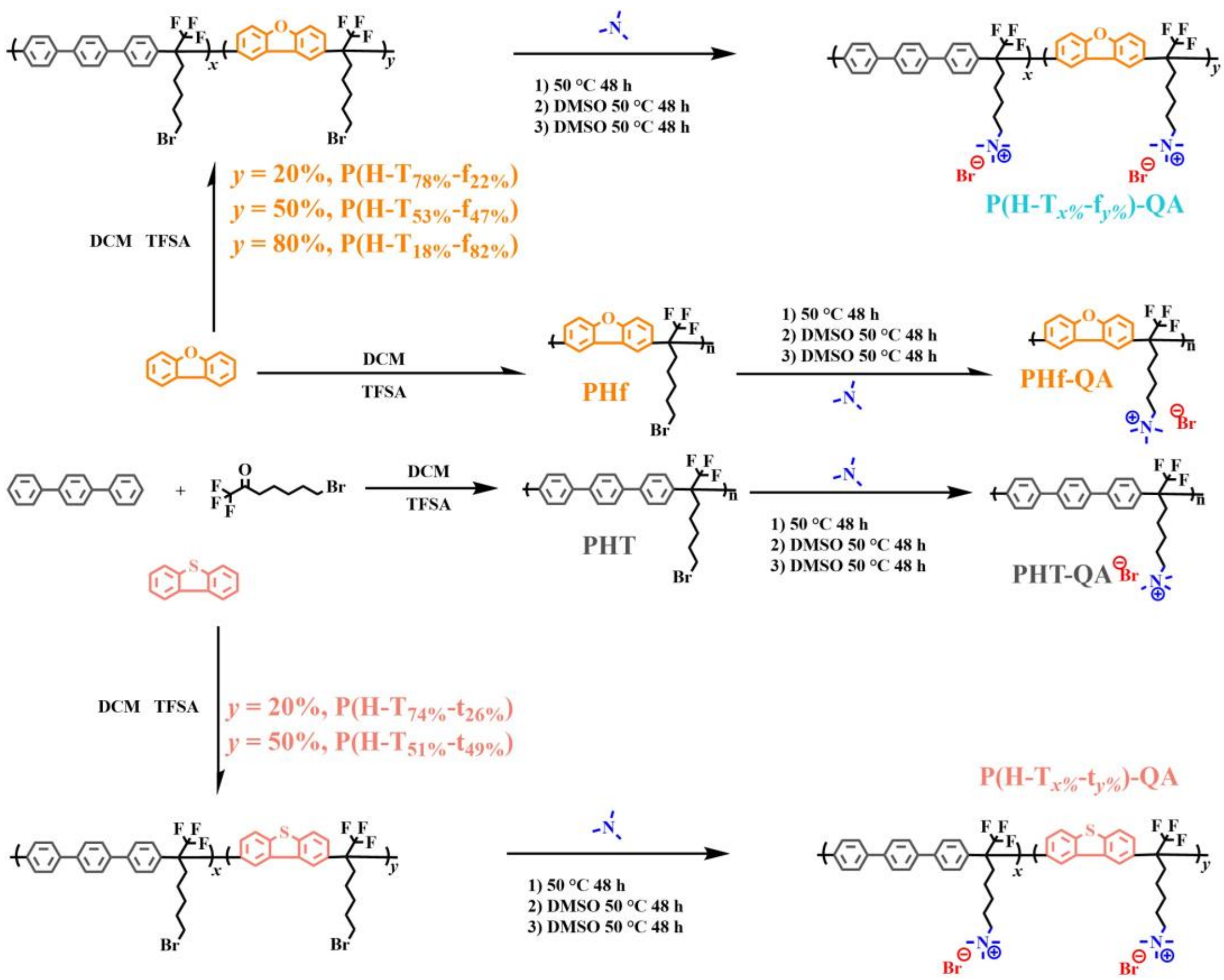

Figure S1. Synthesis pathways to the various polymers.

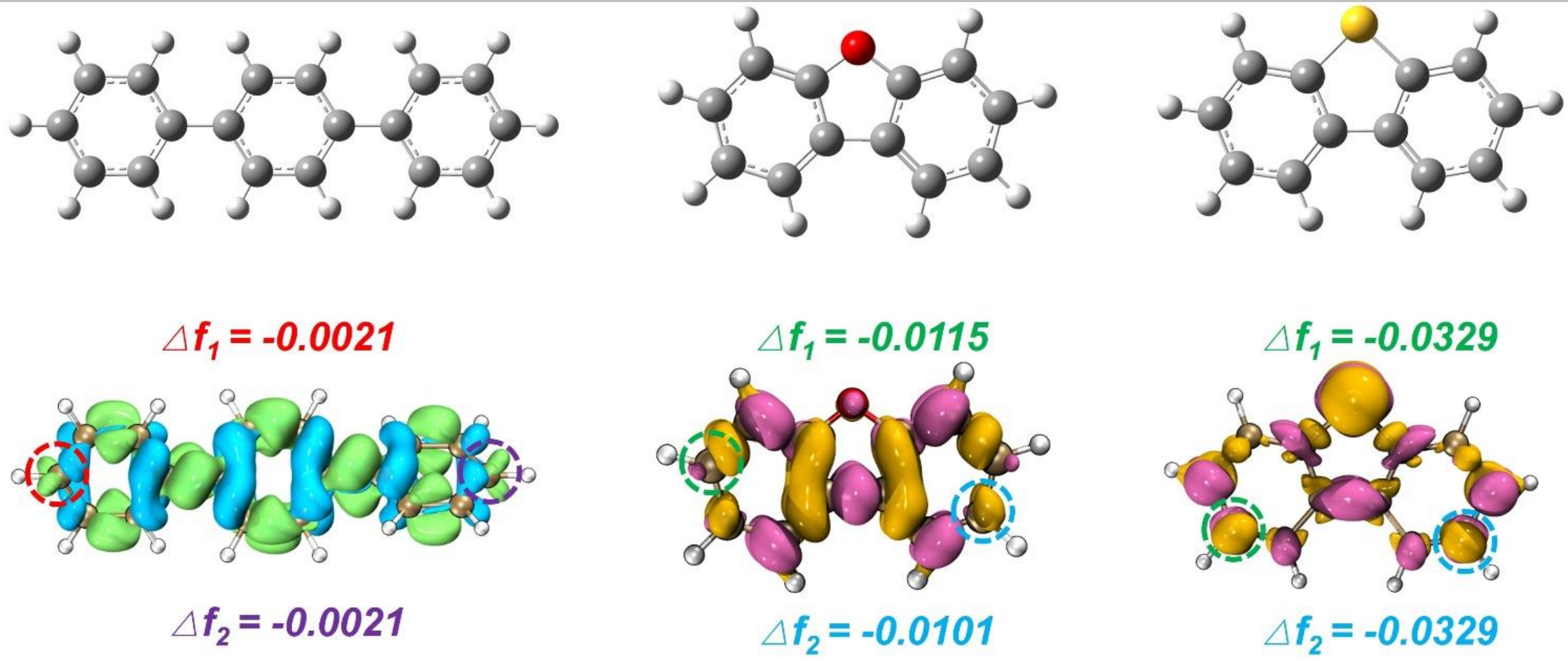

Figure S2. Molecular structures and dual descriptors of the different monomers.

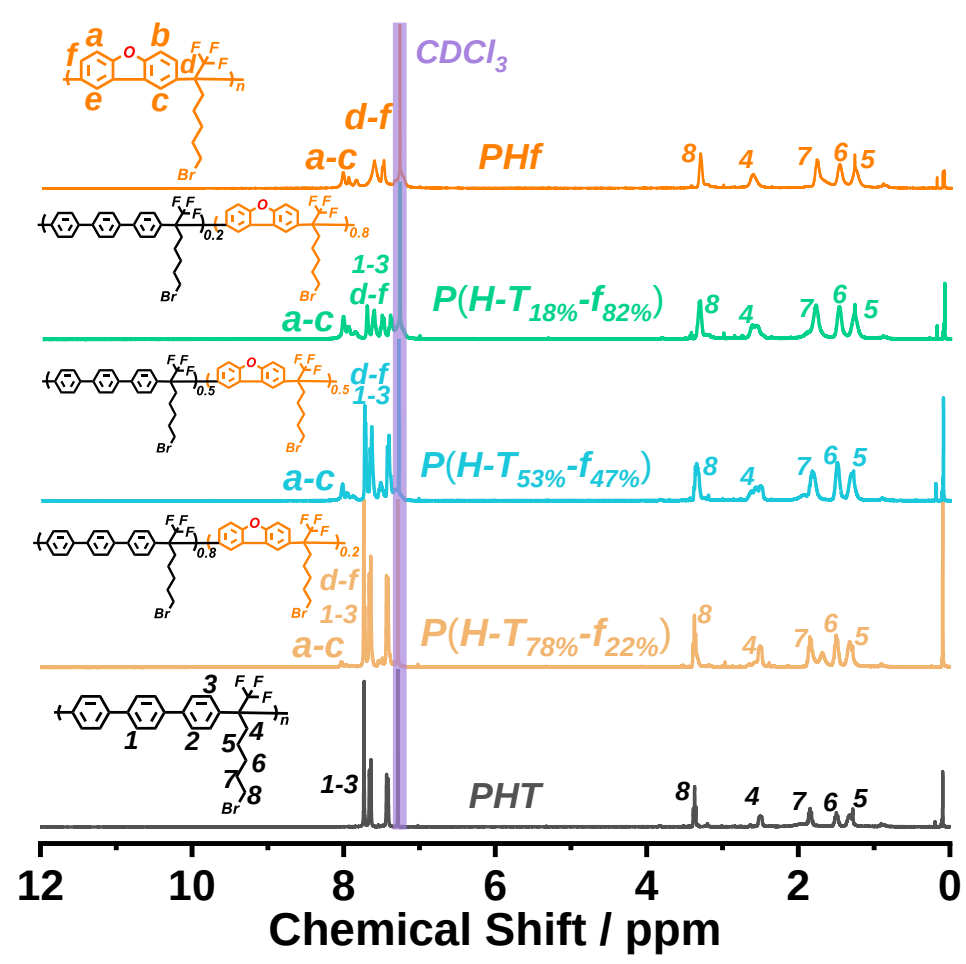

Figure S3. ^{1}H NMR spectra of PHT, P(H-T_x-f_y) and PHf.

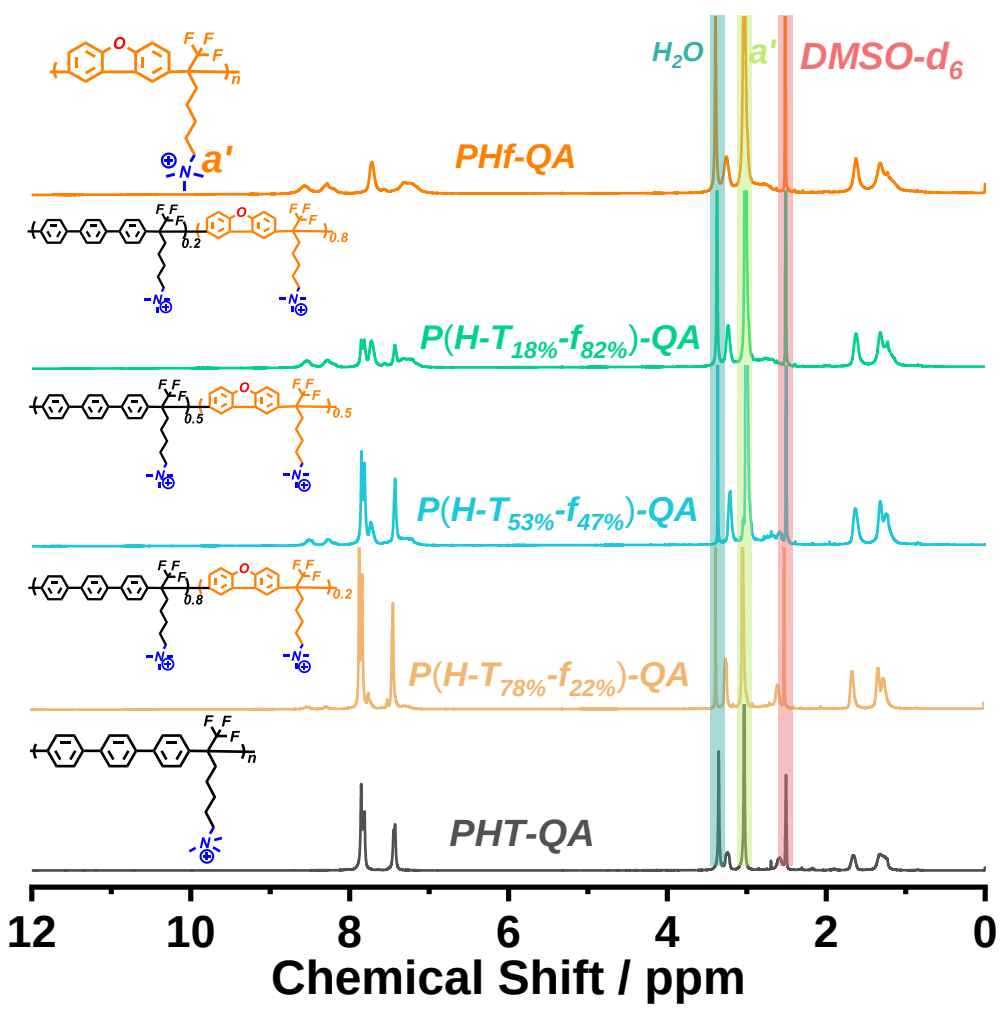

Figure S4. ^{1}H NMR spectra of PHT-QA, P(H-T_x-f_y)-QA and PHf-QA.

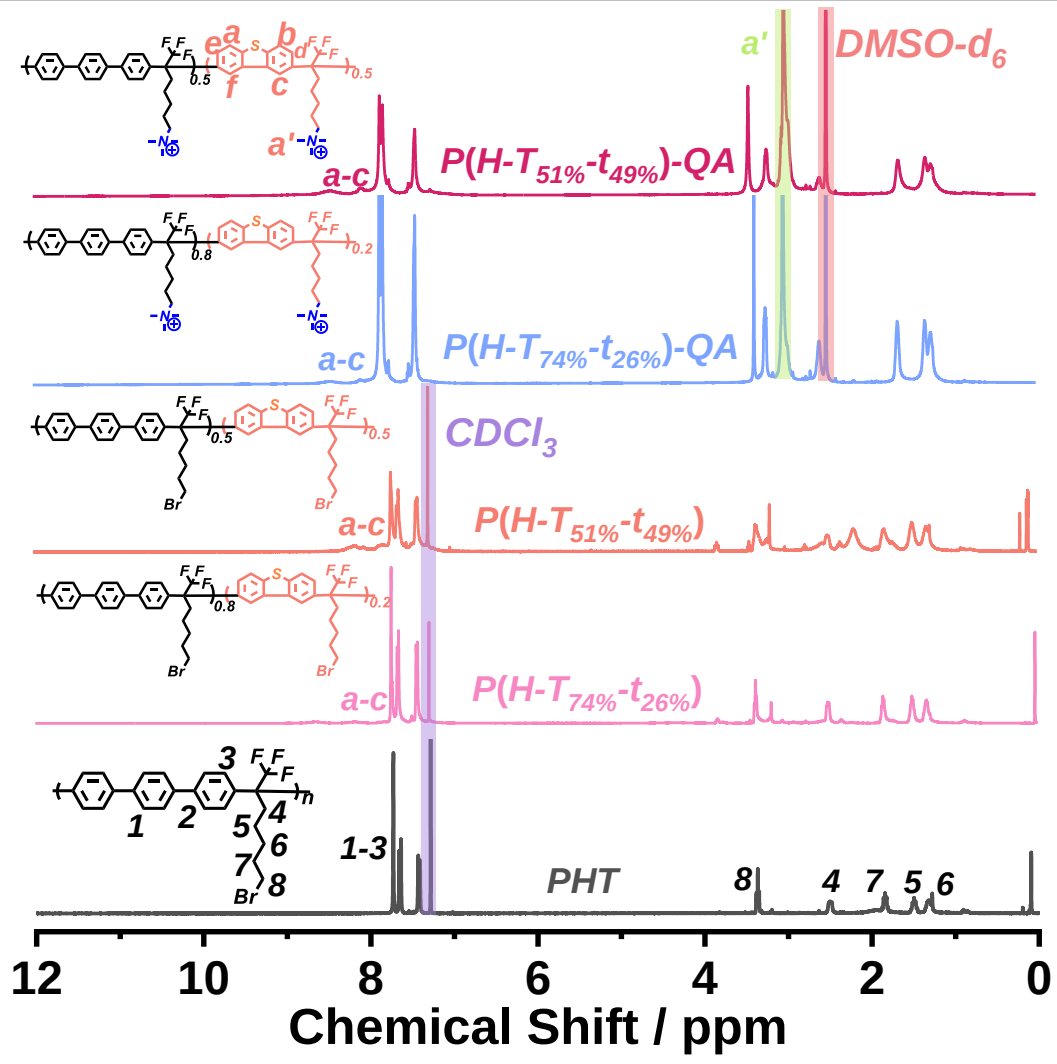

Figure S5. ^{1}H NMR spectra of PHT-QA, P(H-T_x-t_y) and P(H-T_x-t_y)-QA.

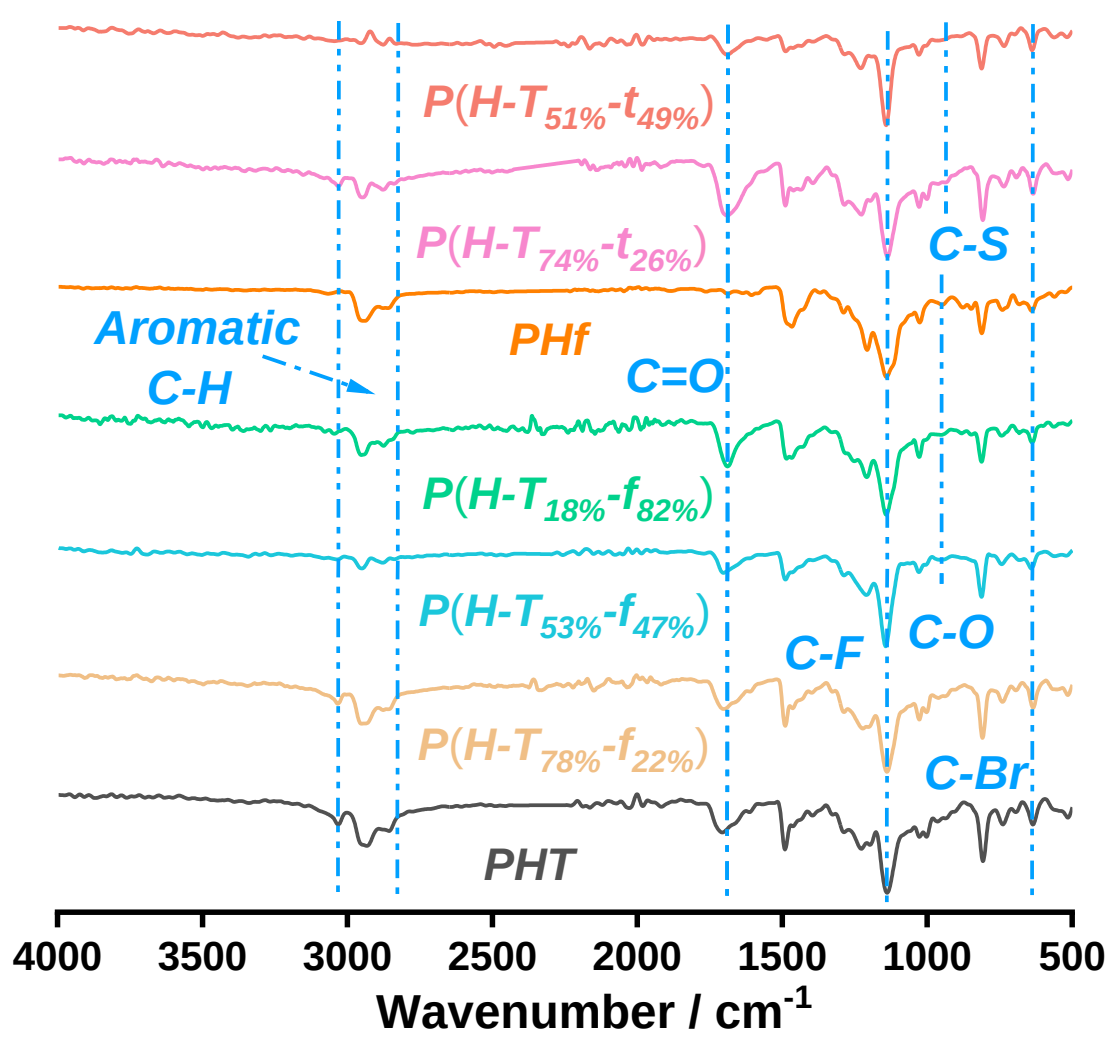

Figure S6. FTIR spectra of PHT, P(H-T_x-f_y), P(H-T_x-t_y) and PHf.

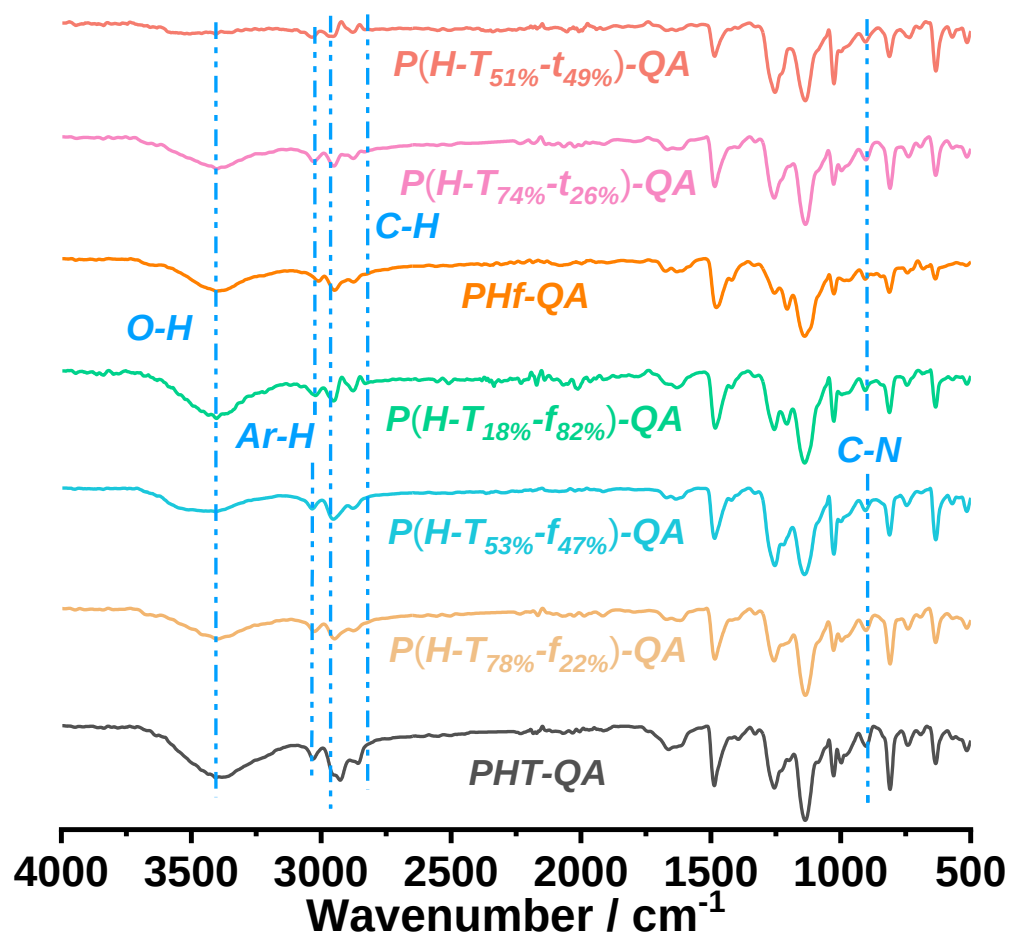

Figure S7. FTIR spectra of PHT-QA, P(H-T_x-f_y)-QA, P(H-T_x-t_y)-QA and PHf-QA.

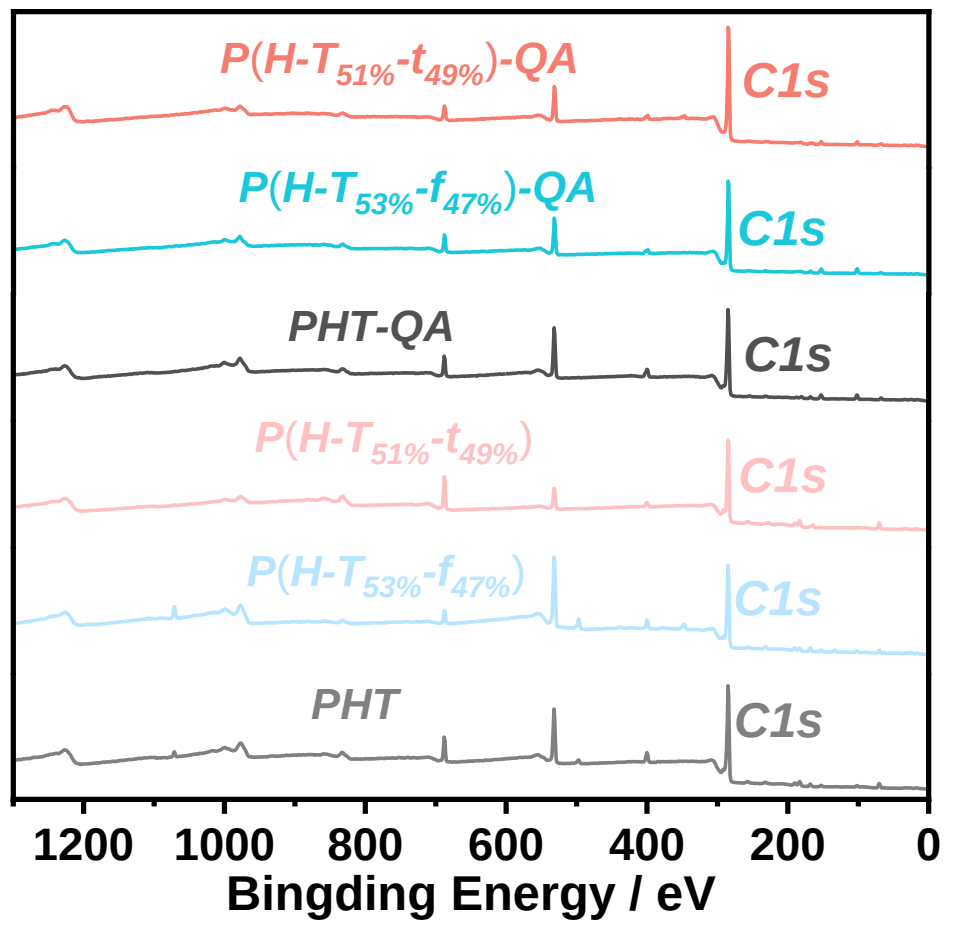

Figure S8. Wide-scan XPS survey spectrum of PHT, P(H-$T_{53\%}$-$f_{47\%}$) and P(H-$T_{51\%}$-$t_{49\%}$) and their quaternized derivatives.

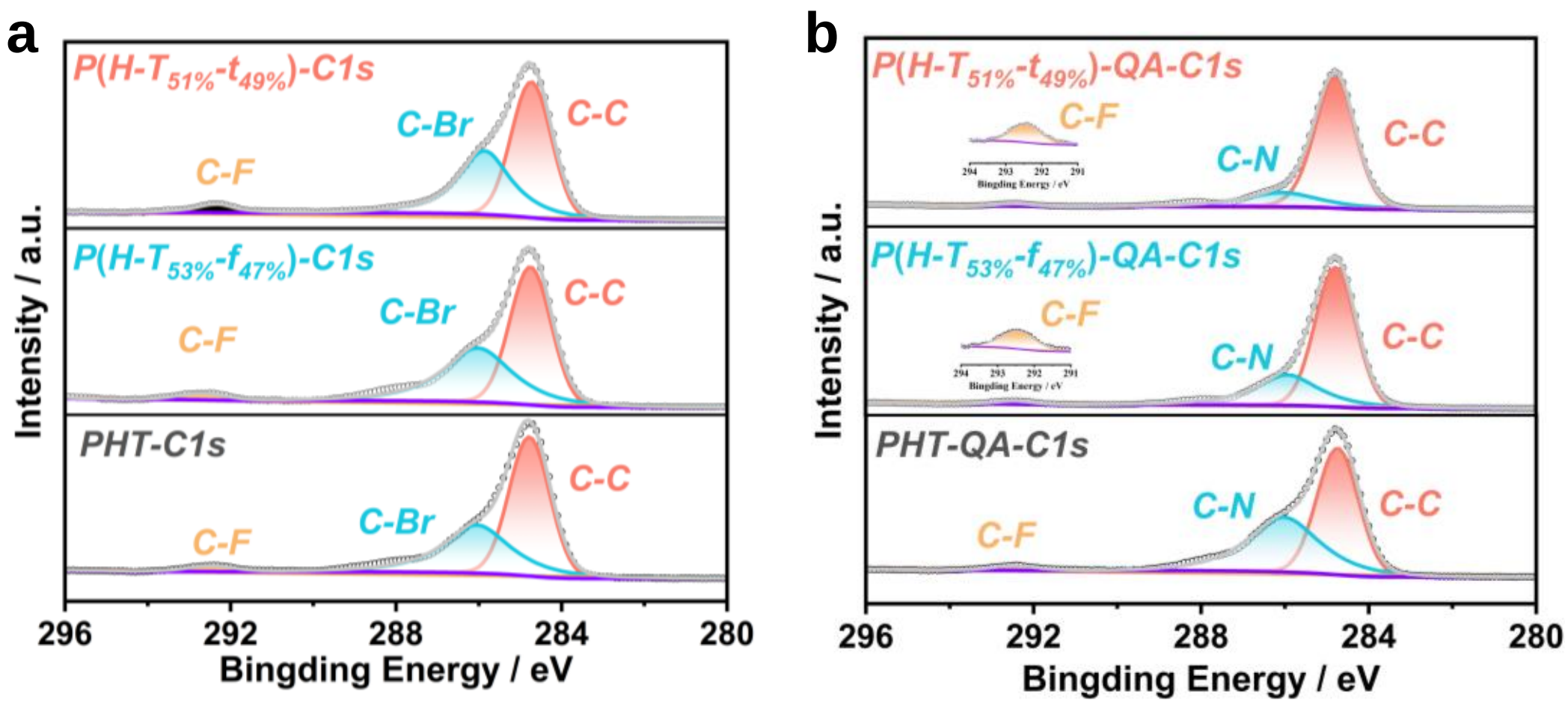

Figure S9. (a) XPS-C1s of PHT, P(H-$T_{53\%}$-$f_{47\%}$) and P(H-$T_{51\%}$-$t_{49\%}$), and (b) their quaternized derivatives.

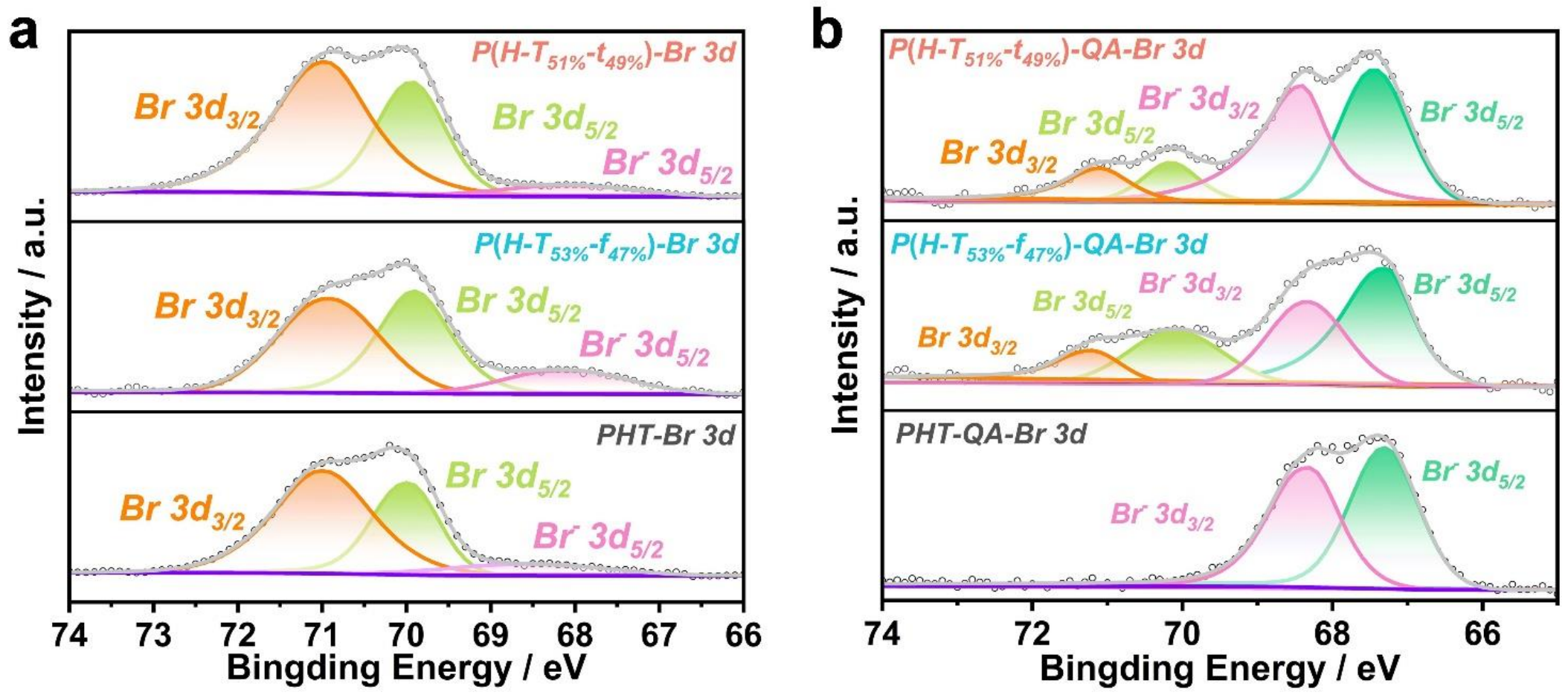

Figure S10. (a) XPS-Br3d of PHT, P(H-$T_{53\%}$-$f_{47\%}$) and P(H-$T_{51\%}$-$t_{49\%}$), and (b) their quaternized derivatives.

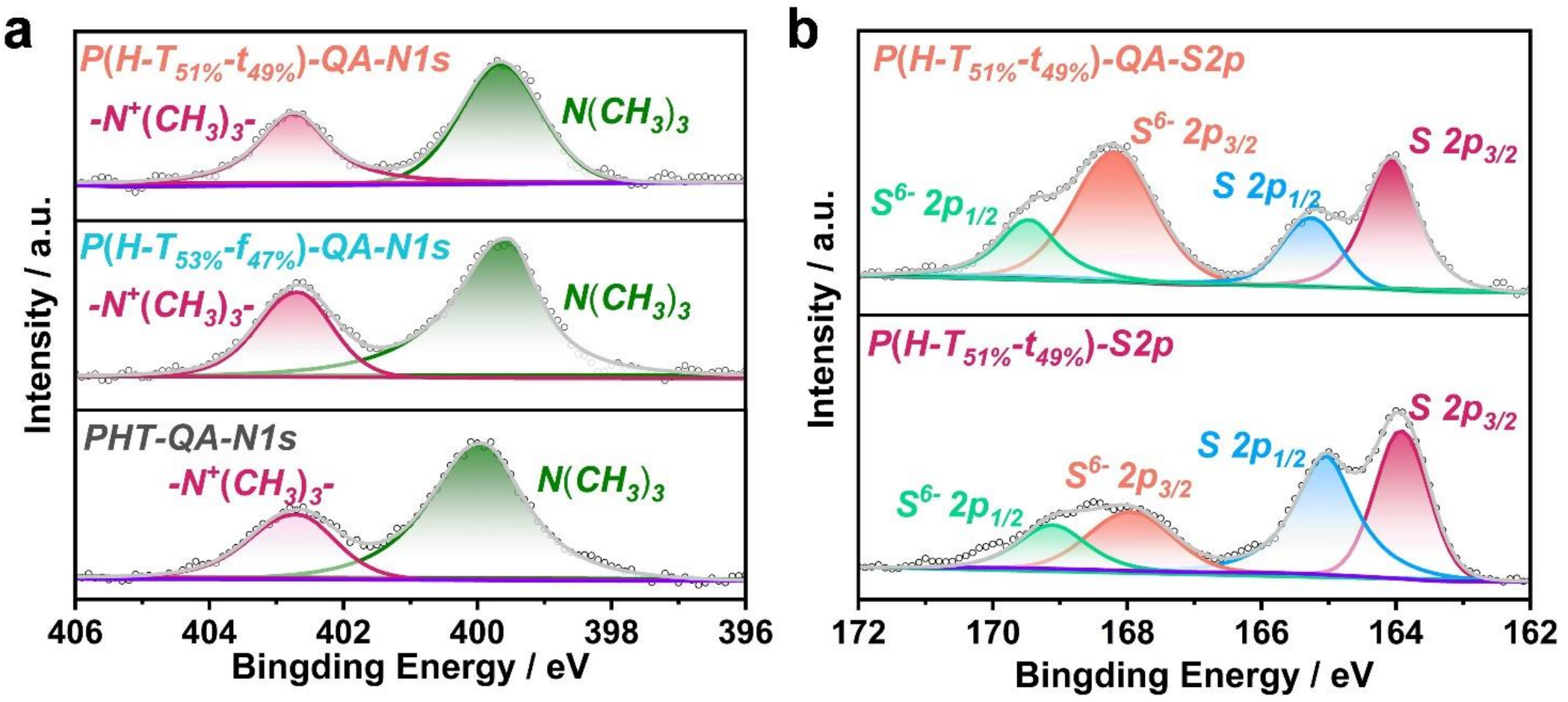

Figure S11. (a) XPS-N1s of PHT-QA, P(H-$T_{53\%}$-$f_{47\%}$)-QA and P(H-$T_{51\%}$-$t_{49\%}$)-QA; (b) XPS-S2p of P(H-$T_{51\%}$-$t_{49\%}$) and P(H-$T_{51\%}$-$t_{49\%}$)-QA.

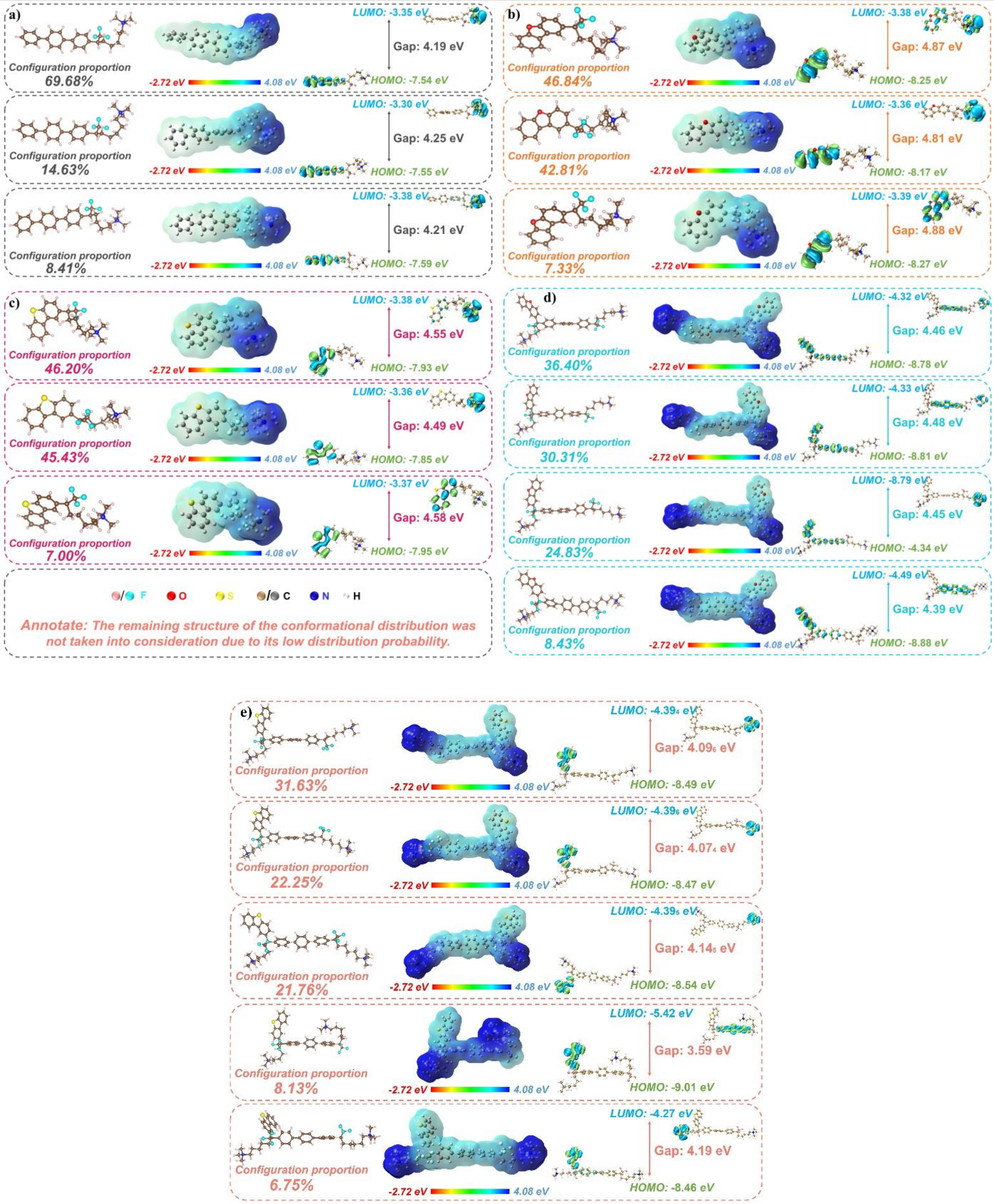

Figure S12. Molecular configurations, surface electrostatic potentials (ESPs) distributions and HOMO-LUMO energy levels of (a) PHT-QA, (b) PHf-QA, (c) PHt-QA, (d) P(H-$T_{50\%}$-$f_{50\%}$)-QA and (e) P(H-$T_{50\%}$-$t_{50\%}$)-QA

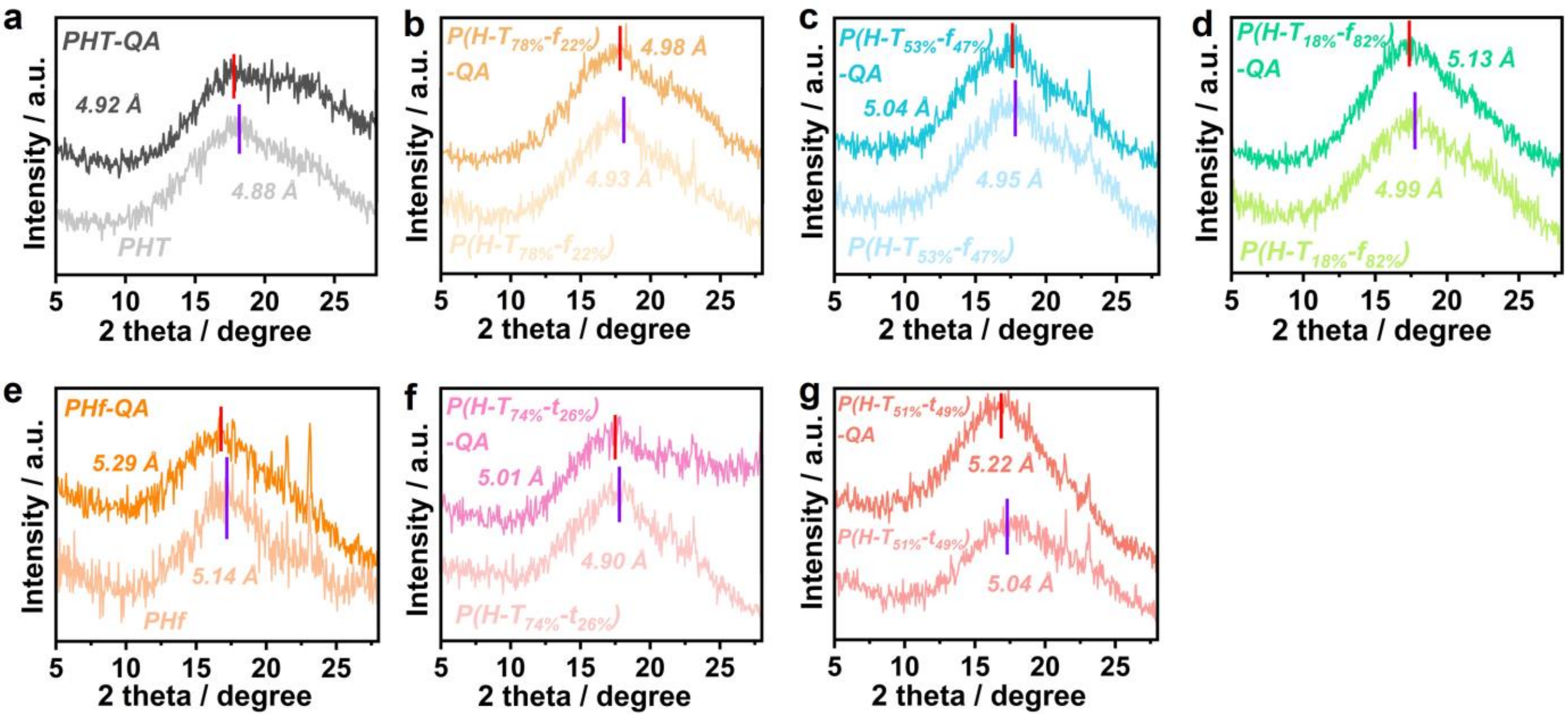

Figure S13. XRD patterns of (a) PHT-QA, (b) P(H-$T_{78\%}$-$f_{22\%}$)-QA, (c) P(H-$T_{53\%}$-$f_{47\%}$)-QA, (d) P(H-$T_{18\%}$-$f_{82\%}$)-QA, (e) PHf-QA, (f) P(H-$T_{74\%}$-$t_{26\%}$)-QA, and (g) P(H-$T_{51\%}$-$t_{49\%}$)-QA.

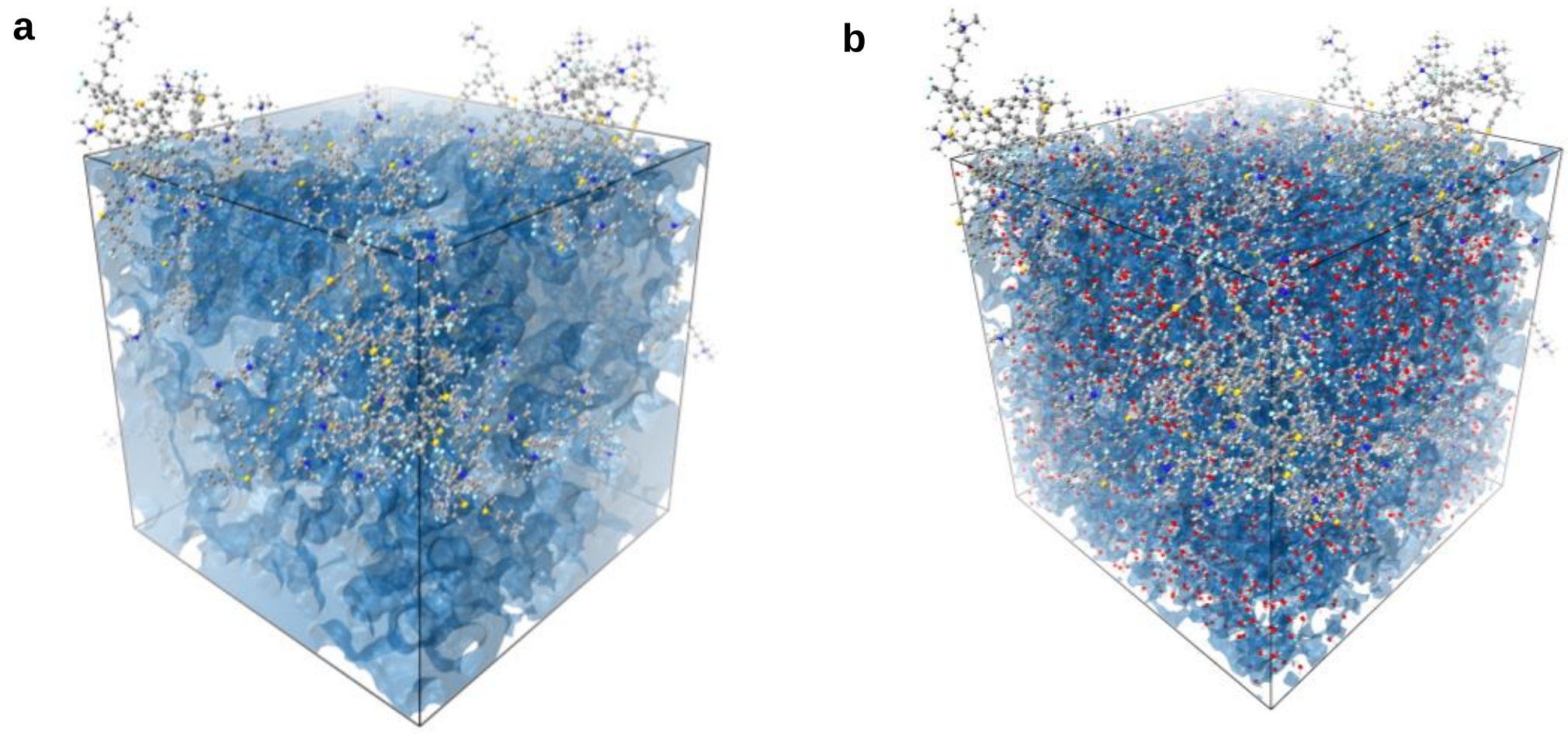

Figure S14. MD simulation results of PHt-QA membranes in the (a) dry and (b) wet state.

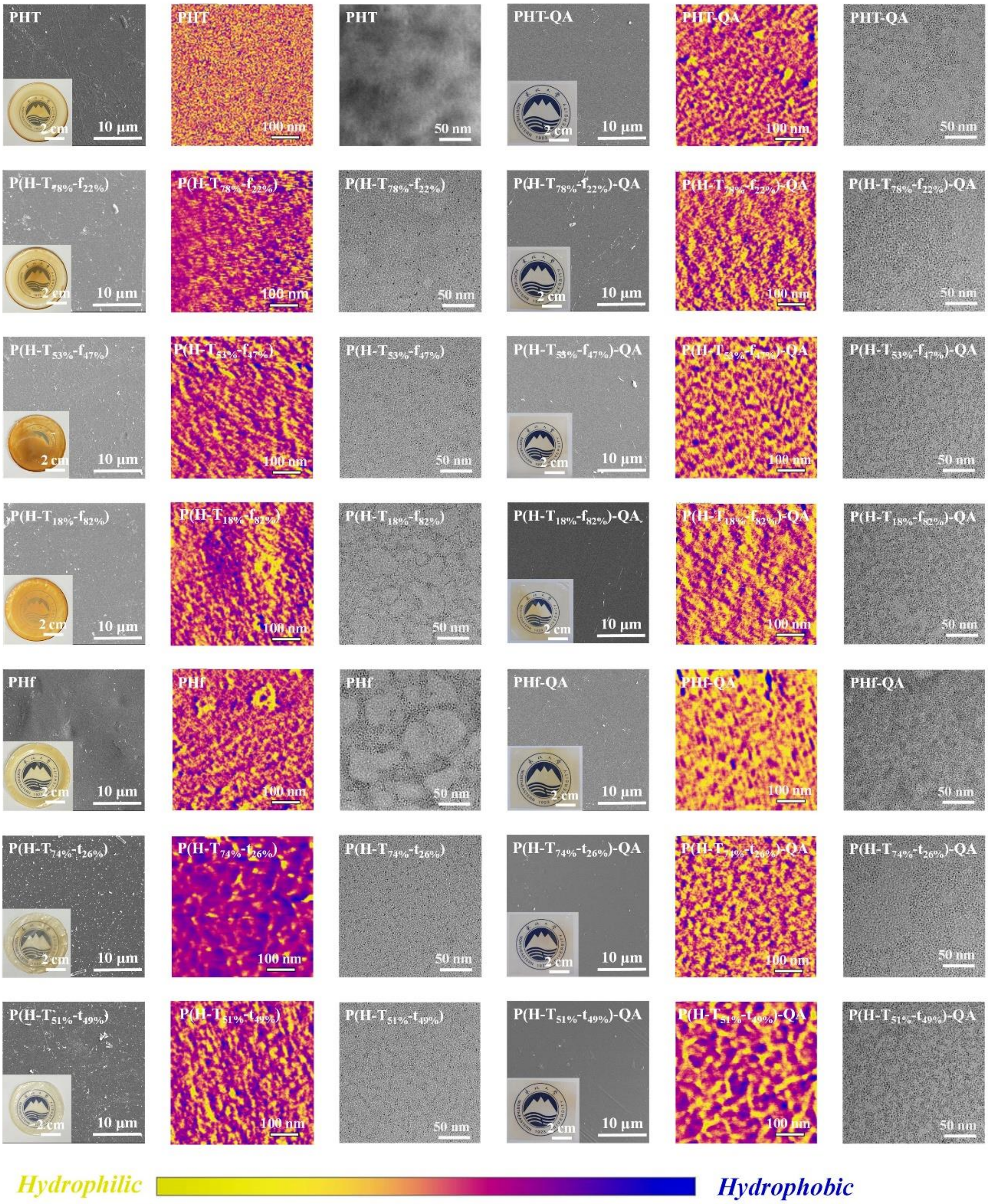

Figure S15. SEM and digital images (inserts), as well as AFM and TEM images of the various membranes. The color bands represent the different phases in the AFM phase diagrams.

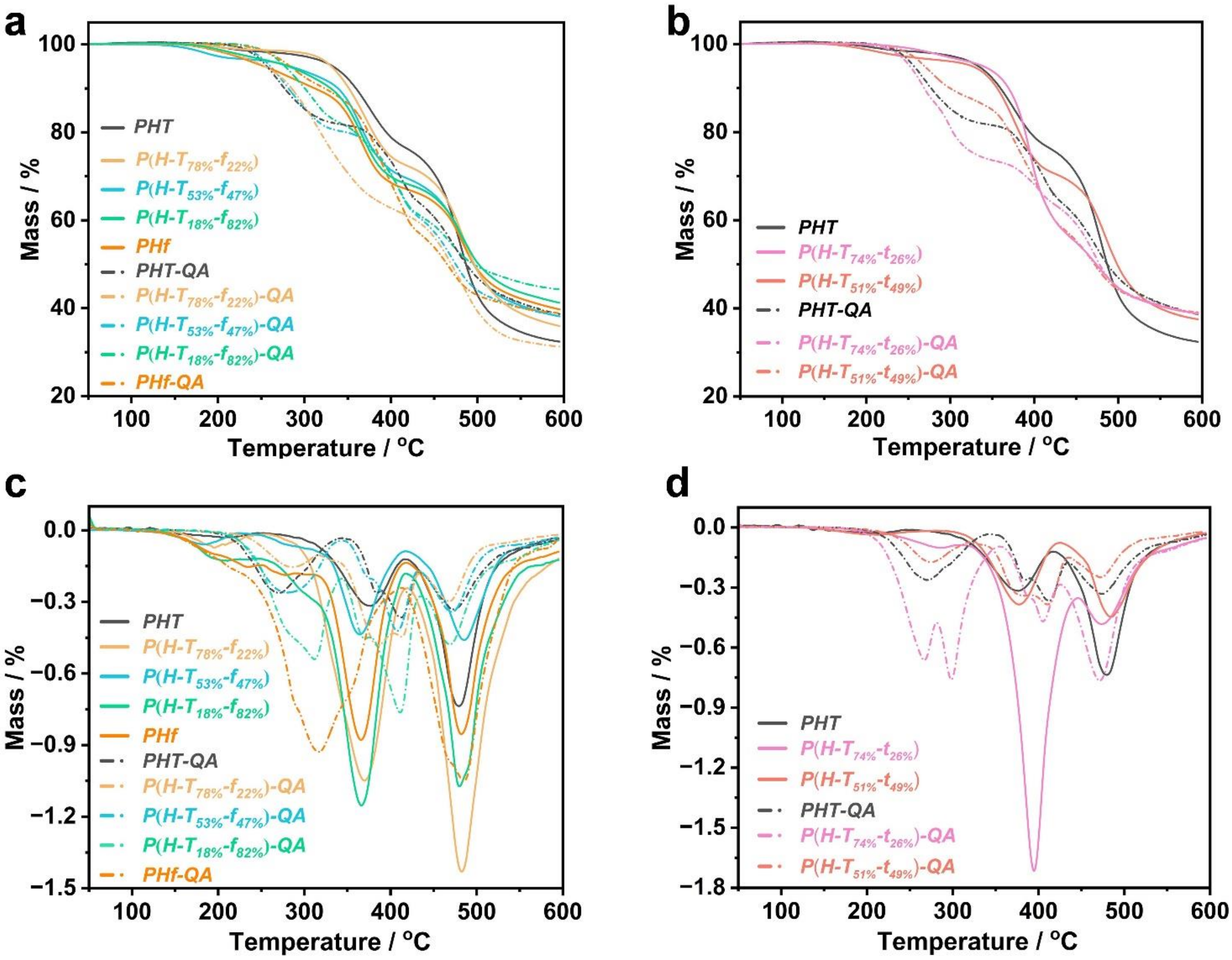

Figure S16. TGA and DTG curves of the various membranes recorded under N_2 at a heating rate of 10 °C min^{-1}.

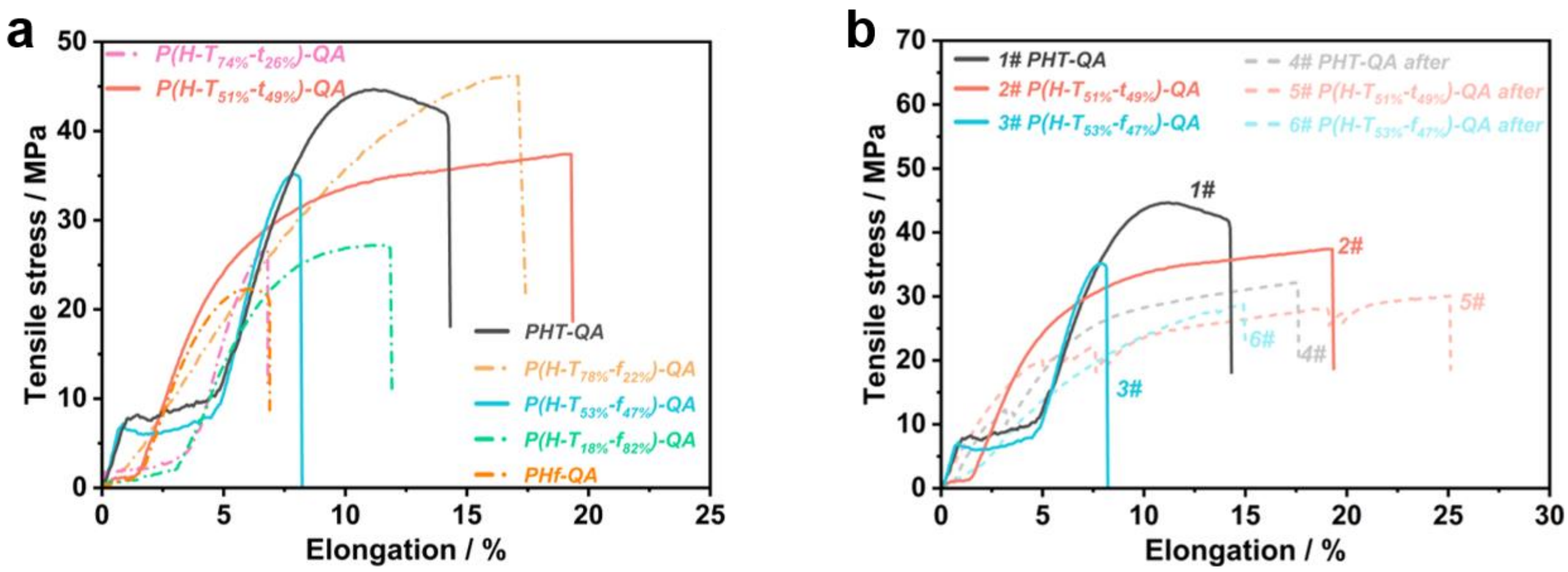

Figure S17. (a) Tensile stress-strain curves of various membranes; and (b) mechanical stress-strain curves of the three representative membranes before and after alkaline stability test in 1 M KOH at 80 °C.

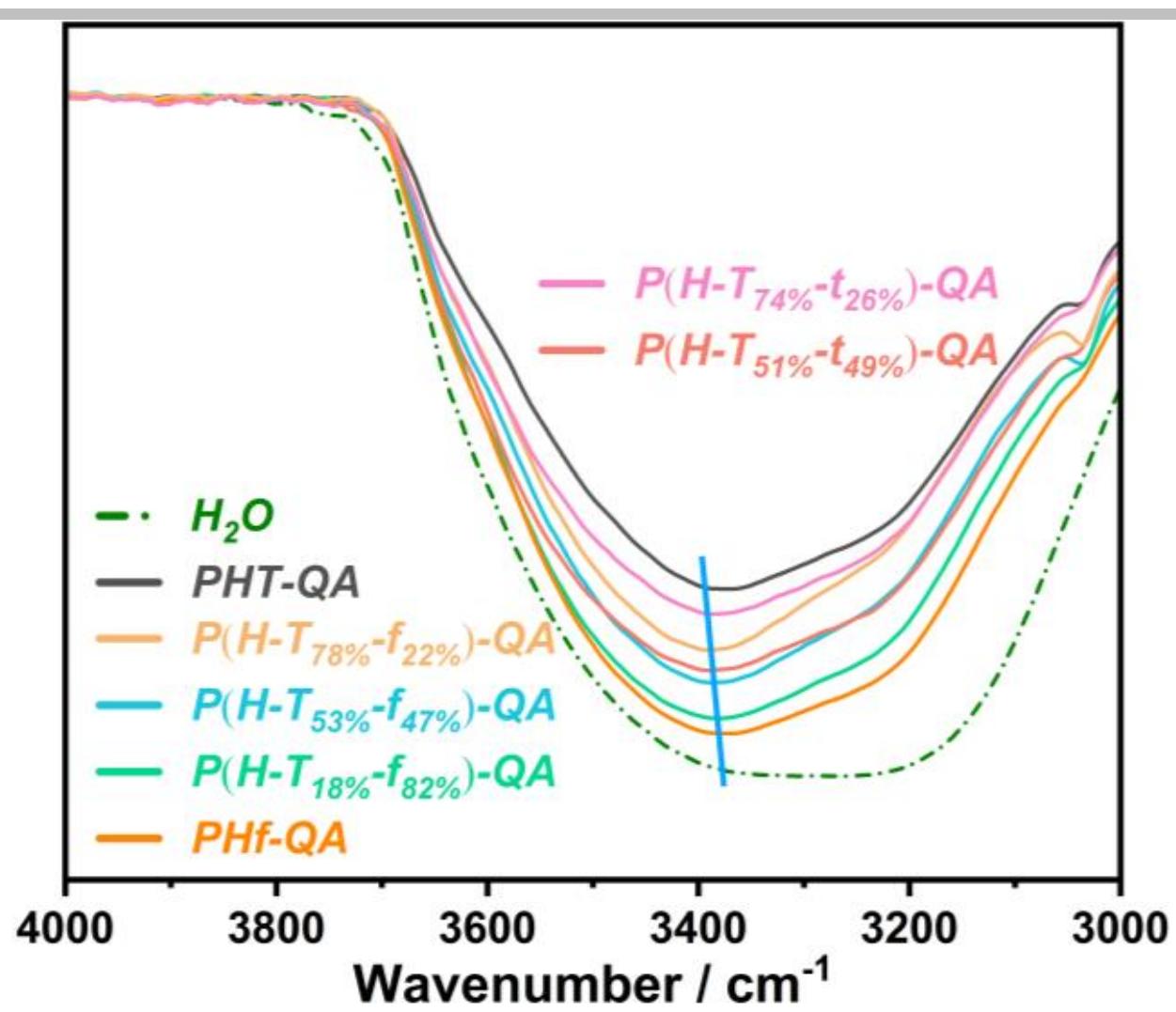

Figure S18. FTIR spectra of wet AEMs and H_2O.

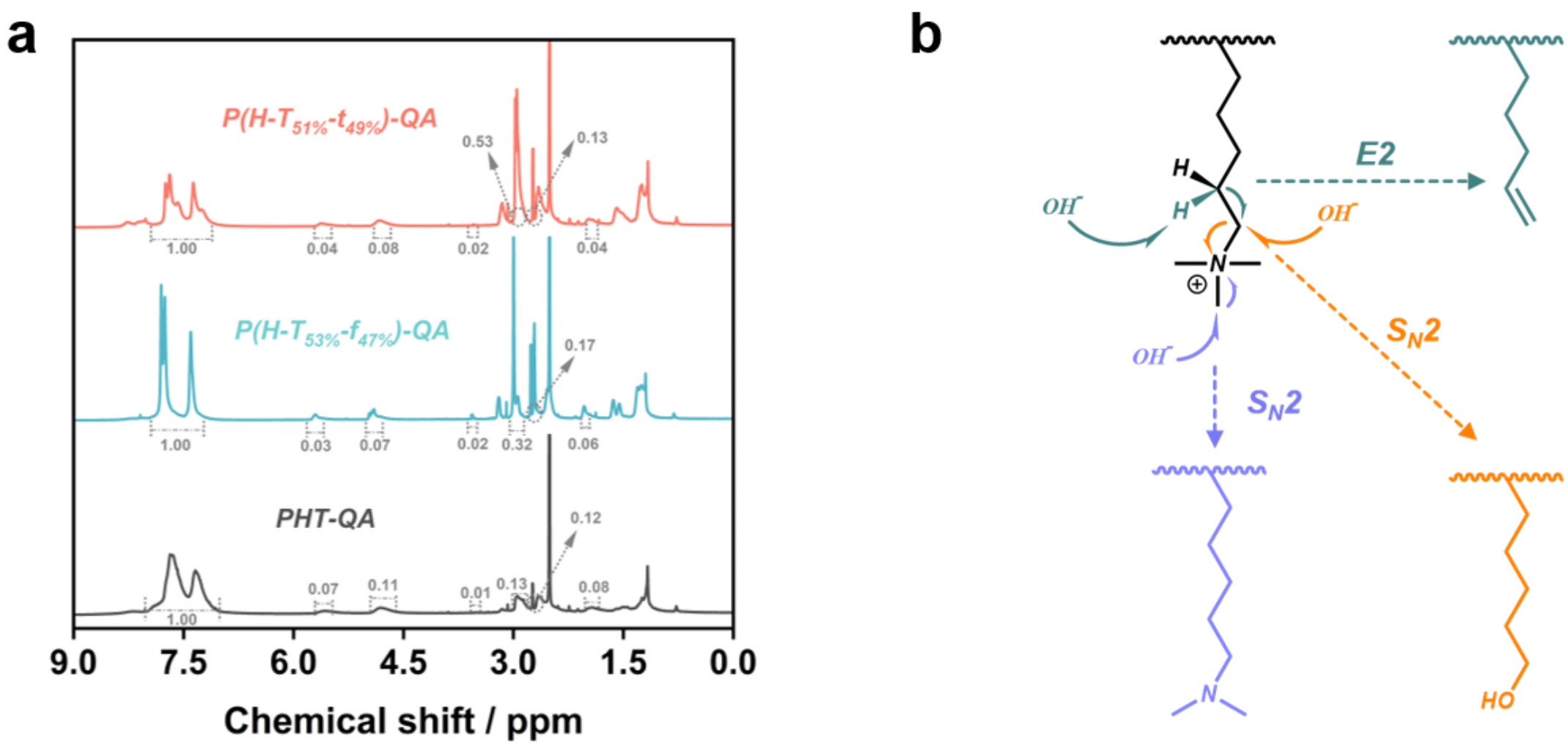

Figure S19. (a) ^{1}H NMR spectra after alkaline stability test of the three representative membranes in 5 M KOH solution at 80 °C for 240 h; (b) possible degradation pathways of the side-chain tetramethylammonium group.

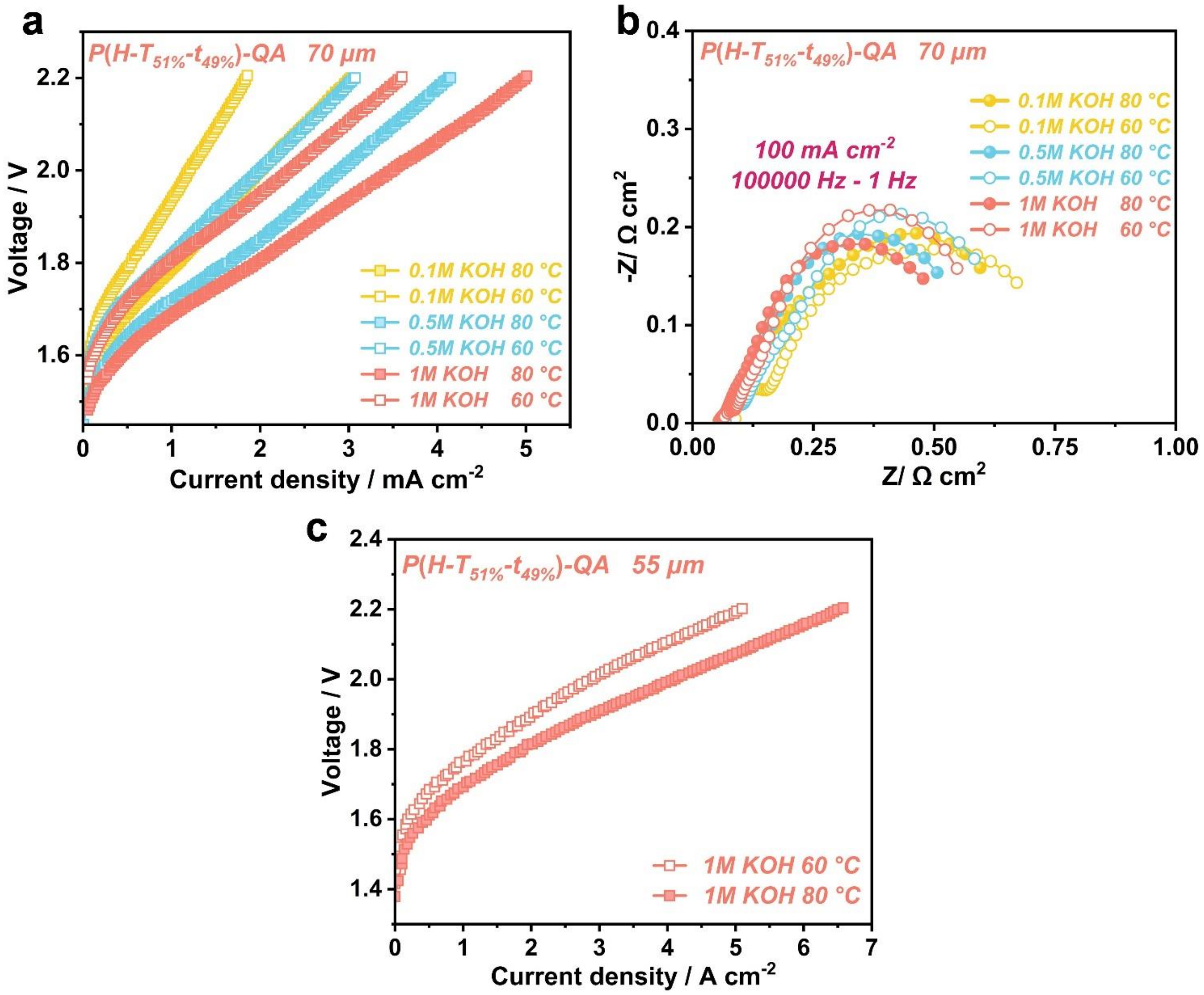

Figure S20. (a) Polarization curves of electrolytic cells assembled with P(H-$T_{51\%}$-$t_{49\%}$)-QA membranes (70 μm) and nonprecious metal catalysts in 1 M aqueous KOH, measured at 60 and 80 °C, respectively. (b) EIS spectrum of AEM in the range of 100000 Hz-1Hz recorded under the same conditions at 100 mA cm^{-2}, (c) polarization curves of P(H-$T_{51\%}$-$t_{49\%}$)-QA (55 μm) in the electrolytic cell assembled with non-precious metal catalysts and recorded under the same conditions.

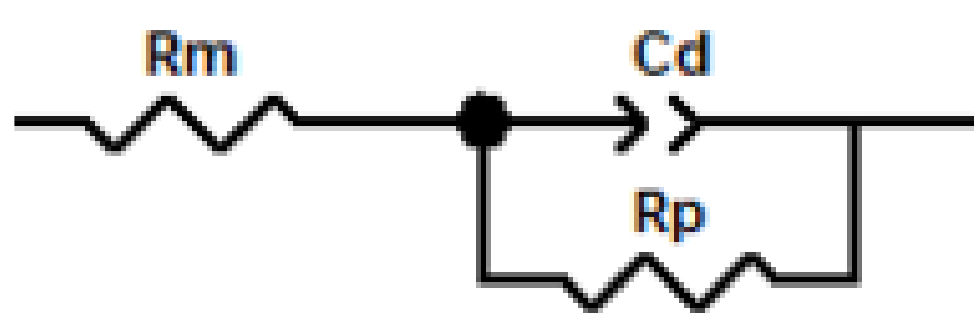

Figure S21. Equivalent circuit model of the two-electrode full cell.

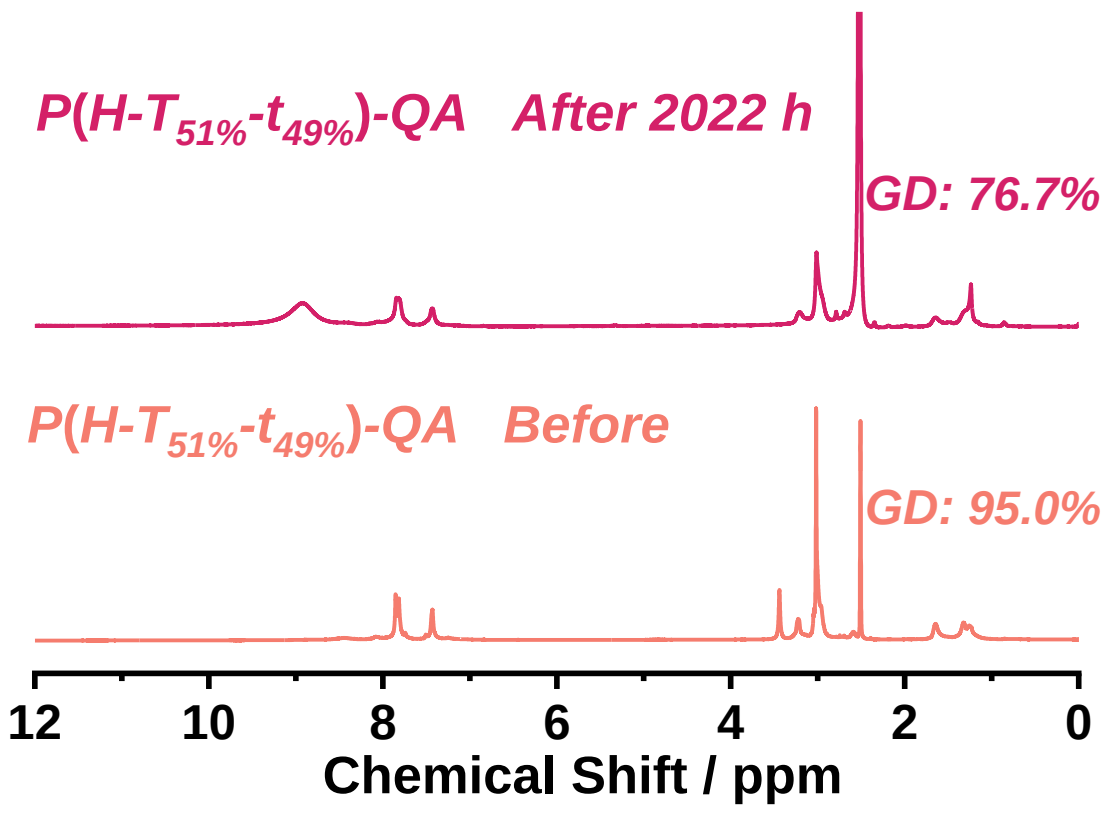

Figure S22. ^{1}H NMR spectra of P(H-$T_{51\%}$-$t_{49\%}$)-QA (70 μm) measured before and after the durability test.

Table S1. MD simulation of the corresponding values related to the free volume fraction.

Membrane	PHT-QA	PHf-QA	PHt-QA	P(H-$T_{50\%}$-$f_{50\%}$)-QA	P(H-$T_{50\%}$-$t_{50\%}$)-QA
Without water	68.25%	71.51%	71.17%	69.83%	69.80%
With water	34.85%	34.99%	35.23%	34.98%	35.37%

Table S2. IEC, water uptake, swelling and water contact angle data of the AEMs.

Membranes	IEC (mmol g^{-1})		Swelling 30 ℃		Water uptake (%)	Hydration number (λ)	Water contact angle
	Theoretical	Experimental	Area (%)	Volume (%)			
PHT-QA	1.93	1.91±0.02	17.3±1.3	32.3±2.8	18.1±0.9	5.23	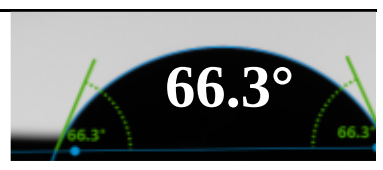
P(H-$T_{78\%}$-$f_{22\%}$)-QA	1.98	1.82±0.04	15.5±2.6	24.2±3.1	21.1±1.1	6.41	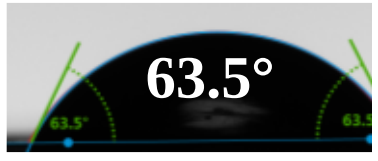
P(H-$T_{53\%}$-$f_{47\%}$)-QA	2.05	1.88±0.06	11.5±1.7	27.4±3.5	25.0±2.0	7.38	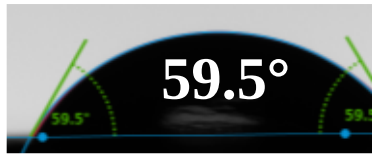
P(H-$T_{18\%}$-$f_{82\%}$)-QA	2.13	1.97±0.05	10.8±0.7	14.3±0.8	28.9±1.4	8.17	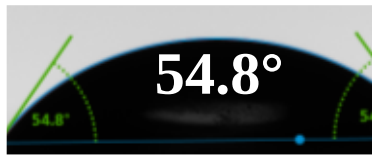
PHf-QA	2.19	2.09±0.04	26.9±2.1	44.4±2.7	33.8±1.2	9.03	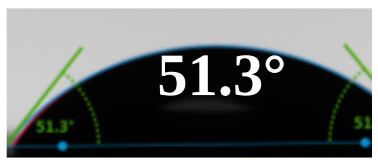
P(H-$T_{74\%}$-$t_{26\%}$)-QA	1.96	1.95±0.01	16.2±0.9	22.8±1.9	21.9±0.5	6.26	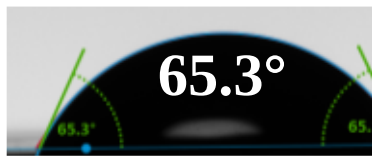
P(H-$T_{51\%}$-$t_{49\%}$)-QA	2.02	1.92±0.02	14.5±0.3	20.9±1.0	26.3±1.6	7.52	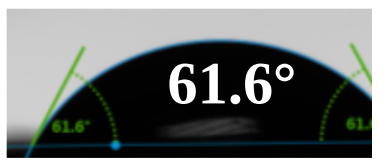

Table S3. Molecular-weight characteristics of representative polymers determined by GPC.

Polymer	PHT	P(H-$T_{53\%}$-$f_{47\%}$)	P(H-$T_{51\%}$-$t_{49\%}$)
M_n / kDa	196	215	190
M_w / kDa	208	241	210
PDI (M_w/M_n)	1.06	1.11	1.12

Table S4. Resistance changes of the cells assembled with P(H-$T_{53\%}$-$f_{47\%}$)-QA and P(H-$T_{51\%}$-$f_{49\%}$)-QA before and after AEMWE durability test.

Resistance (Ω cm^2)	P(H-$T_{53\%}$-$f_{47\%}$)-QA (60 μm)		P(H-$T_{51\%}$-$t_{49\%}$)-QA (60 μm)	
	0 h	410 h	0 h	578 h
R_m	0.050	0.105	0.049	0.060
R_p	0.195	0.385	0.275	0.294

References

[1] J. Martínez-Araya, "The dual descriptor potential", *Journal of Mathematical Chemistry* **62** (2024): 1094-1112.

[2] M. Frisch, G. Trucks, H. Schlegel, G. Scuseria, M. Robb, J. Cheeseman, G. Scalmani, V. Barone, G. Petersson, H. Nakatsuji, X. Li, M. Caricato, A. Marenich, J. Bloino, B. Janesko, R. Gomperts, B. Mennucci, H. Hratchian, J. Ortiz, A. Izmaylov, J. Sonnenberg, D. Williams-Young, F. Ding, F. Lipparini, F. Egidi, J. Goings, B. Peng, A. Petrone, T. Henderson, D. Ranasinghe, V. Zakrzewski, J. Gao, N. Rega, G. Zheng, W. Liang, M. Hada, M. Ehara, K. Toyota, R. Fukuda, J. Hasegawa, M. Ishida, T. Nakajima, Y. Honda, O. Kitao, H. Nakai, T. Vreven, K. Throssell, J. Montgomery, Jr., J. Peralta, F. Ogliaro, M. Bearpark, J. Heyd, E. Brothers, K. Kudin, V. Staroverov, T. Keith, R. Kobayashi, J. Normand, K. Raghavachari, A. Rendell, J. Burant, S. Iyengar, J. Tomasi, M. Cossi, J. Millam, M. Klene, C. Adamo, R. Cammi, J. Ochterski, R. Martin, K. Morokuma, O. Farkas, J. Foresman, D. Fox, Gaussian 16, Revision B.01, Gaussian, Inc., Wallingford CT, 2016.

[3] T. Lu, F. Chen, "Multiwfn: A multifunctional wavefunction analyzer", *Journal of Computational Chemistry* **33** (2012): 580-592.

[4] T. Lu, "A comprehensive electron wavefunction analysis toolbox for chemists, Multiwfn", *Journal of Chemical Physics* **161** (2024): 082503.

[5] S. Spicher, S. Grimme, Robust atomistic modeling of materials, organometallic, and biochemical systems", *Angewandte Chemie International Edition* **59** (2020): 15665-15673.

[6] T. Lu, Molclus program, version 1.12, 2025.

[7] C. Bannwarth, S. Ehlert, S. Grimme, "GFN2-xTB-An accurate and broadly parametrized self-consistent tight-binding quantum chemical method with multipole electrostatics and density-dependent dispersion contributions", *Journal of Chemical Theory and Computation* **15** (2019): 1652-1671.

[8] N. O'Boyle, M. Banck, C. James, C. Morley, T. Vandermeersch, G. Hutchison, "Open Babel: An open chemical toolbox", *Journal of Cheminformatics* **3** (2011): 33.

[9] T. Lu, Q. Chen, Simple, "Efficient, and universal energy decomposition analysis method based on dispersion-corrected density functional theory", *Journal of Physical Chemistry A* **127** (2023): 7023-7035.

[10] W. Humphrey, A. Dalke, K. Schulten. "VMD: Visual molecular dynamics", *Journal of Molecular Graphics and Modelling* **14** (1996): 33-38.
